# Structural Elucidation of Malonylcommunol and 6β-Hydroxy-*trans*-communic Acid, Two Undescribed Diterpenes from *Salvia cinnabarina*. First Examples of Labdane Diterpenoids from a Mexican *Salvia* Species

**DOI:** 10.3390/molecules25081808

**Published:** 2020-04-15

**Authors:** Celia Bustos-Brito, Antonio Nieto-Camacho, Simón Hernandez-Ortega, José Rivera-Chávez, Leovigildo Quijano, Baldomero Esquivel

**Affiliations:** Instituto de Química, Universidad Nacional Autónoma de México, Circuito Exterior, Ciudad Universitaria, Mexico City 04510, Mexico; celia.bustos@iquimica.unam.mx (C.B.-B.); anieto@unam.mx (A.N.-C.); simonho@unam.mx (S.H.-O.); jrivera@iquimica.unam.mx (J.R.-C.)

**Keywords:** *Salvia cinnabarina*, labdane diterpenoid, α-glucosidase

## Abstract

The aerial parts of *Salvia cinnabarina* afforded two undescribed labdane diterpenoids **1** and **2** (malonylcommunol and 6β-hydroxy-*trans*-communic acid) along with two known labdane diterpenoids, *trans*-communic acid (**3**) and *trans*-communol (**4**). Additionally, seven known metabolites were also isolated; two isopimarane diterpenoids **5** and **6**, two sesquiterpenoids identified as β-eudesmol (**7**) and cryptomeridiol (**8**), and three aromatic compounds identified as phthalic acid (**9**), a mixture of tyrosol fatty acid esters (**10**) and the flavone salvigenine (**11**). While compounds compounds **1**–**3** showed significant inhibition of yeast α-glucosidase, compounds **2**, **3** and **7** had no anti-inflammatory activity in the edema model induced by TPA. This paper is not only the first report on a wild population of *Salvia cinnabarina*, but also of the presence of labdane-type diterpenoids in a Mexican *Salvia* sp.

## 1. Introduction

*Salvia* L. is the largest genus of Lamiaceae family of plants, with over 1000 species distributed worldwide [1]. The name of the genus derives from the Latin verb “salvare” which means to heal. This is very likely due to the fact that some of these plant species (*Salvia divinorum* Epling and Játiva, *S. milthiorrhiza* Bunge and *S. officinalis* L. [2]) have been used since ancient times to treat several ailments, and are important medicinal herbs in both traditional and modern medicine. Mexico is one of the most important areas of diversification of the genus in the world, with over 319 species, representing ca. 32% of the total, although this number is continuously increasing due to the discovery of new species [3]. Several *Salvia* species are utilized in different regions of the country for treatment of various ailments, with some well recognized as part of several medicinal plant complexes [2].

*Salvia cinnabarina* M. Martens and Galeotti (Section Incarnatae), a plant originating from Mexico, is used for the treatment of colic and rheumatism in the Mexican state of Chiapas. Antibacterial and spasmolytic activities have been also described for this species [4].

Previous studies on *S. cinnabarina* include the analysis of volatile organic compounds [5], and the essential oil obtained from fresh aerial parts of the plant by steam distillation [6], as well as phytochemical analysis of the leaf exudate leading to the isolation of a 3,4-*seco*-isopimarane diterpenoid whose structure and relative stereochemistry was established as 3,4-*seco*-isopimara-4(18),7,15-trien-3-oic acid (**12**) by spectroscopic and X-ray diffraction analysis [7,8]. Compound **12** has been tested in several in vitro and in vivo models and exhibits a wide array of biological activities, such as antispasmodic in the isolated guinea-pig ileum model, inhibition of urinary bladder contractility in rats [9] and intestinal motility in mice [10]. Hypotensive activity in a rat model [11], along with anxiolytic and anti-depressive effects in the elevated plus-maze and the forced swimming tests in mice have also been described [12]. Compound **12** showed antimutagenic activity in the Ames test on *Salmonella typhimurium* and *Escherichia coli* [13] and also had an anticlastogenic effect in human lymphocytes of its sodium salt [14]. It should be noted that, up to now, all the studies on *S. cinnabarina*, have been carried out solely on cultivated material from different Botanical Gardens.

Continuing with our systematic study of the genus *Salvia* in Mexico, and ongoing investigation for biological activity diterpenes of chemosystematic importance [15], we report herein the first study on a wild population of *S. cinnabarina* collected in the State of Puebla (Mexico). Several diterpenoids of the labdane (**1**–**4**) and isopimarane (**5**–**6**) skeletons were isolated, as well as two eudesmane-type sesquiterpenoids (**7**–**8**), phtalic acid (**9**), tyrosol derivatives (**10**) and the flavone salvigenin (**11**). Compounds **1**–**2** proved to be undescribed labdane-type diterpenoids related to *trans*-communic acid (**3**) and *trans*-communol (**4**), also isolated from this plant, and their structures were established as malonylcommunol (**1**) and 6β-hydroxy-*trans*-communic acid (**2**). While compounds **1**–**3** showed significant inhibition of yeast α-glucosidase, compounds **2**,**3** and **7** showed no anti-inflammatory activity in the edema model induced by TPA.

## 2. Results and Discussion

**Figure 1:**
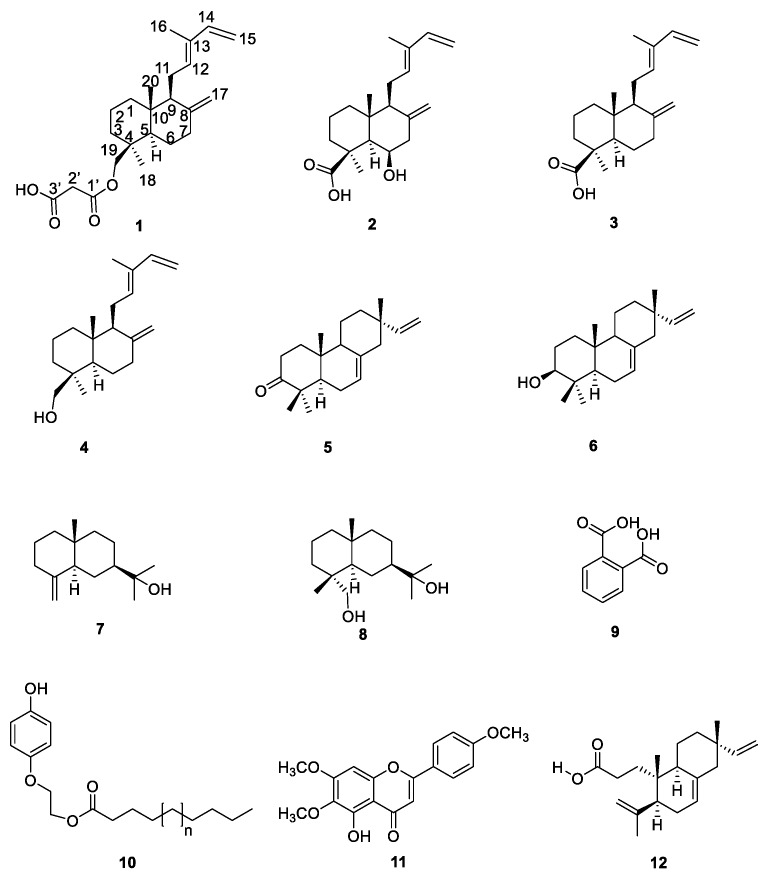
Chemical structures of **1**–**12**.

The aerial parts of *Salvia cinnabarina* afforded 11 compounds (Figure 1) after extensive chromatographic separation and purification.

Compound **1** was isolated as a solid, m.p. = 85–90 °C. The mass spectrum obtained by DART technique allowed to establish the chemical formula as C_23_H_34_O_4_ with seven degrees of unsaturation. The ^13^C NMR spectrum (Table 1) of **1**, corroborated the presence of 23 carbon atoms, which, according to the HSQC spectrum correspond to ten methylenes (two sp^2^ and eight sp^3^), four methines (two sp^2^ and two sp^3^), two quaternary carbons, four nonprotonated carbons and three methyl groups. In the ^13^C NMR spectrum of **1** (Table 1), signals for an exocyclic methylene, such as the one present at C-8:C-17 of *trans*-communic acid (**3**) [16] and *trans*-communol (**4**) [16] are observed at *δ* 147.7 (C) and 108.2 (CH_2_) ppm. Observed signals for carbon atoms of a terminal vinyl group at *δ* 141.7 (CH) and 110.1 (CH_2_), together with those observed at 133.8 (CH), 133.7 (C), 23.3 (CH_2_) and 12.0 (CH_3_), suggested that compound **1** has a side chain identical to the one present in diterpenes **3** and **4**. Therefore, the signals at δ 141.7 and 110.1 were assigned to C-14 and C-15 and those at 133.8, 133.7, 23.3 and 12.0 *ppm*, to C-12, C-13, C-11 and C-16, respectively. The chemical shifts of C-14 (141.7) and C-16 (12.0) confirmed the configuration of the C-12:C-13 double bond as *E* (*trans*). The chemical shifts of these carbon atoms are very sensitive to the double bond configuration, being observed at approximately 130 and 20 *ppm* in the case of a *Z* (*cis*) configuration [17,18].

The ^1^H NMR spectrum of **1** (Table 1), was also similar to that of *trans*-communol (**4**), with signals for the terminal vinyl group being observed at *δ* 6.32 (1H, dd, *J* = 17.3 and 10.7 Hz, H-14), 5.04 (1H, d, *J* = 17.4 Hz, H-15 *trans*) and 4.88 (1H, d, *J* = 10.7 Hz, H-15b *cis*). A triplet at 5.39 *ppm* (*J* = 6.2 Hz) was assigned to H-12 and a singlet for three hydrogen atoms at 1.74 *ppm* to the C-16 methyl group. Characteristic signals for the hydrogen atoms of the exocyclic methylene at C-8 (H-17) were also observed in the ^1^H NMR spectrum of **1** as broad singlets at 4.82 and 4.47 *ppm*.

**Figure 2:**
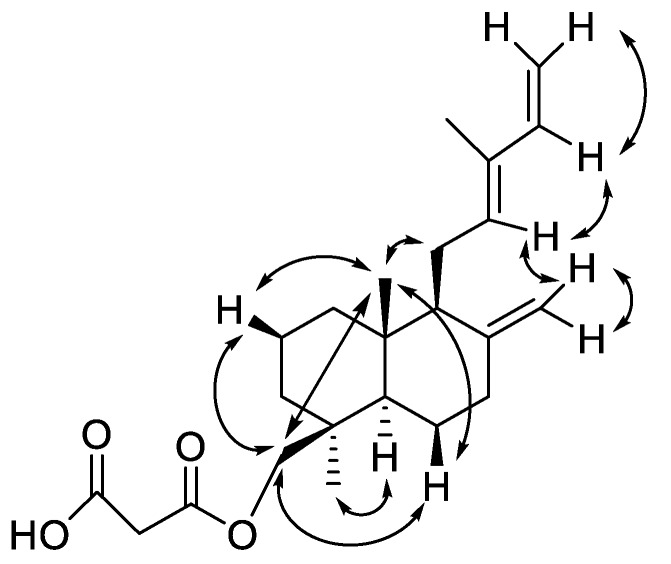
Key NOESY interactions observed for compound **1**.

**Figure 3:**
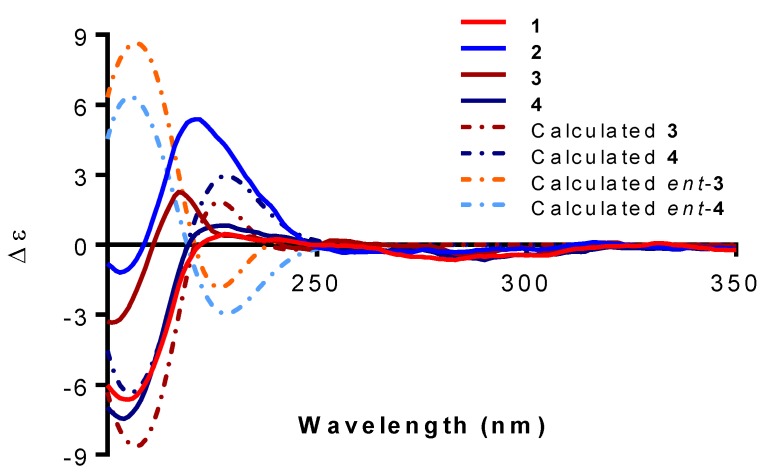
Experimental ECD spectra for compounds **1**–**4**. Calculated ECD spectra for **3** (burgundy dashed line), *ent*-**3** (orange dashed line), compound **4** (navy dashed line) and *ent*-**4** (sky-blue dashed line). A negative shift of −20 nm was required to match the spectra.

A relevant signal in the ^1^H NMR spectrum for the structural assignment of diterpene **1**, was a broad singlet integrating for two hydrogen atoms at 3.41 *ppm*, that disappears upon addition of D_2_O. This signal correlated in the HMBC spectrum (Table 1) with two carbonyl signals located at 169.6, 168.3 and a methylene signal at 68.5 *ppm*. The first two signals are assigned to the carbonyls of an acid and an ester, respectively, and the third to a methylene whose hydrogen atoms are observed in the ^1^H NMR spectrum, as an AB system at 4.38 and 3.95 *ppm* (*J* = 10.9). The IR spectrum of **1** is congruent with the existence of a carboxylic acid and an ester group in this compound, since a broad band centered at approximately 3000 cm^−1^ a carbonyl band in 1721 cm^−1^ (characteristic of a carboxylic acid) and a carbonyl ester band at 1736 cm^−1^ were observed. The above discussion, and the similarity between the NMR spectra of *trans*-communol (**4**) and those of **1,** allows us to conclude the presence of a malonic acid ester at position C-19 in compound **1**, which was named malonylcommunol (**1**). The exchange of the protons of the methylene group at 3.41 *ppm* of the malonyl group upon addition of D_2_O could be explained by the enolization of the 1,3 dicarbonyl moiety [19]. The NOESY spectrum of **1** confirms the structure and relative stereochemistry proposed for this unpublished diterpene isolated from *S. cinnabarina*, on account of observation of expected interactions, illustrated in Figure 2. A malonate ester of a labdane diterpenoid from *Calceolaria corymbosa* Ruiz and Pav (Scrophulariaceae), with the same connectivity as malonylcommunol (**1**), was isolated in 1993 by Garbarino and Molinari [17]. However, the double bond has a *Z* configuration between carbons C-12 and C-13 in the diterpene from *C. corymbosa*. According to the authors it belongs to the *ent*-labdane series and therefore is a stereoisomer of **1**. To establish the absolute configuration of **1**, its experimental ECD spectrum (Figure 3) was recorded and compared with those registered for compounds **3** and **4** (Figure 3), whose stereochemistry has been previously determined [16]. The ECD spectrum of **1** displayed a negative Cotton effect at 203 nm and a positive one at 226 nm and was in good agreement with those of **3** and **4**. Additionally, ECD calculations for the 4*S*5*R*9*S*10*R* diastereomers of **3** and **4** and their enantiomers (4*R*5*S*9*R*10*S*) were performed, interestingly, the curves matched the calculated for diasteroisomers 4*S*5*R*9*S*10*R* (Figure 3). Thus, the absolute configuration of compound **1** was determined to be 4*S*5*R*9*S*10*R*.

Diterpene malonates are relatively common, several examples and even products in which both acidic functions of malonic acid are esterified with diterpenic alcohols have been described [20,21,22,23,24,25,26,27]. Compound **1** is an unpublished malonate diterpenoid and the first described in any *Salvia* species.

**Figure 4:**
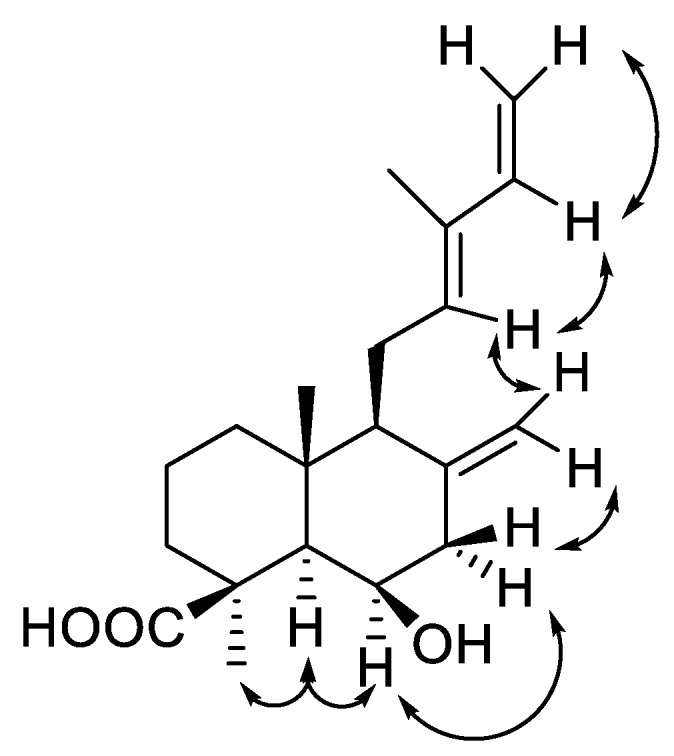
Key NOESY interactions observed for compound **2**.

**Figure 5:**
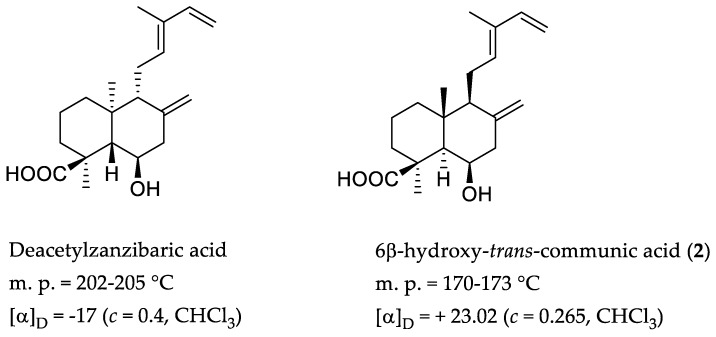
Comparison of some physical properties of deacetylzanzibaric acid with compound **2**.

The second unreported labdane diterpenoid obtained from the dichloromethane extract of *S. cinnabarina* was isolated as a white solid, m.p. 170–173 °C, whose molecular formula established by mass spectrometry as C_20_H_30_O_3_. These data, in addition to the ^1^H and ^13^C NMR data (Table 2), allow us to propose structure **2** for this undescribed diterpene. The ^1^H NMR spectrum exhibits similar signals to those observed for *trans*-communic acid (**3**). The main difference in the spectrum of **2** is the presence of a wide quartet-like signal centered at 4.52 (*J* = 2.1 Hz) *ppm* that is assigned to a hydrogen atom geminal to a hydroxyl group which interacts with a broad singlet at 1.46 *ppm* and with the signals of a methylene at 2.34 and 2.50 *ppm* according to the correlations observed in its COSY spectrum. The signal at 1.46 *ppm* correlates in the HSQC spectrum (Table 2) with a sp^3^ methine carbon observed at 57.5 *ppm* in the ^13^C NMR spectrum of **2** which is assigned by its chemical shift to C-5. These facts allow locating the hydroxyl group at C-6 position and the coupling constants of the observed signal for its geminal hydrogen atom (H-6) indicate a β-axial orientation for the OH group. The analysis of the NOESY spectrum confirms the structure and relative stereochemistry assigned to this product, as the expected correlations are observed and the most relevant being those of H-5 with H-6 and the C-18 methyl (Figure 4). In its IR spectrum characteristic bands for hydroxy groups in 3684, 3590 and 3531 cm^−1^ were observed, as well as a broad band centered at approximately 3000 cm^−1^ attributed to the hydroxy group of a carboxylic acid, whose carbonyl group is observed at 1725 cm^−1^. In the spectrum, signals attributable to double bonds at 1647 and 1605 cm^−1^ were also observed. Based on the previous discussion, product **2** should be named 6β-hydroxy-*trans*-communic acid, which has not been previously described. The absolute configuration of 6β-hydroxy-*trans*-communic acid (**2**) was established to be *4S5R6R9S10R* by comparison of its experimental ECD curve with those recorded for **1**, **3** and **4**, which coexist in this population of *S. cinnabarina*. In addition, in 1965, a diterpene called zanzibaric acid was isolated from *Trachylobium verrucosum* Engl., whose structure and absolute configuration were established by spectroscopic means, as well as chemical correlation with a derivative of neo-abietic acid, establishing that zanzibaric acid is an *ent*-labdane [28]. Treatment of the zanzibaric acid methyl ester with NaOH in ethanol gave a product called 6-deacetylzanzibaric acid, whose connectivity is similar to that found for product **2**. However, comparison of m.p. and the specific rotation indicates that they are diastereoisomeric substances, as indicated in Figure 5.

**Figure 6:**
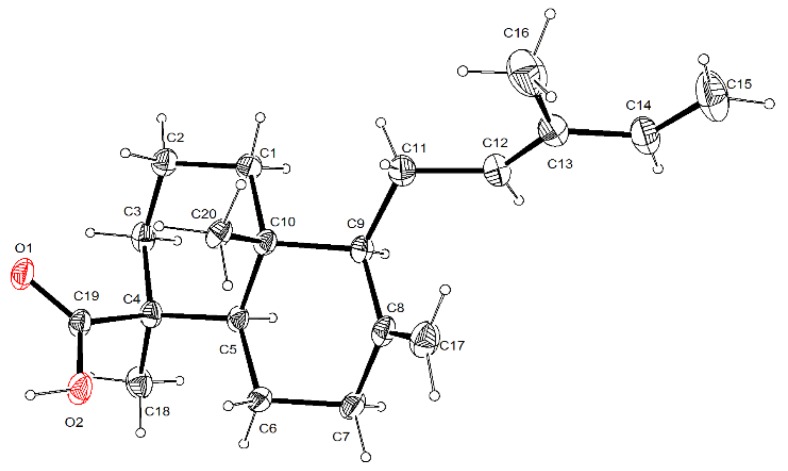
PLUTO plots of the single crystal X-ray diffraction structures of *trans*-communic acid (**3**).

Compound **3,** isolated from this population of *S. cinnabarina,* was identified as *trans*-communic acid based on its spectroscopic properties and comparison with literature data [29]. Even though *trans*-communic acid (**3**) has been obtained from various natural sources [30], it was first isolated from *Juniperus communis* L. (Cupressaceae) [31]. The structure and configuration of this compound were established by extensive chemical transformations of the natural acid, its sodium salt and derivatives, as well as by correlation with labdane-type diterpenes of known configuration such as torulosol and manool [16]. In 1987 Shie-Ming Peng et al. confirmed the structure and relative configuration by X-ray diffraction study of the methyl ester obtained from *trans*-communic acid (**3**), isolated from fresh leaves of *Calocedrus formosana* Florin [32]. In this study, meticulous attempts to crystallize *trans*-communic acid (**3**) were successful and crystals of the natural product which were suitable for X-ray diffraction were obtained. Figure 6 shows the computer-generated projection of the natural enantiomer of *trans*-communic acid (**3**). The absolute configuration was confirmed by calculating the Flack parameter whose value, x = 0.1 (3), confirms the absolute configuration shown in structure **3**. Based on the above data the absolute configuration of *trans*-communic acid (**3**) was stablished as 4*S*5*R*9*S*10*R*.

It is important to point out that labdane diterpenes have been isolated from several other species of the genus *Salvia*, such as *Salvia sclarea* L., *S. officinalis* L., *S. palaestina* Benth., *S. aethiopis* L., *S. yosgadensis* Freyn and Bornm [33], *S. leriaefolia* Benth. [34], *S. rhytidea* Benth. [35] and *S. reuterana* Boiss [36] which grow in Europe or the Middle East. This is the first time that labdane type diterpenoids have been isolated from a sage of the subgenus Calospahce. It is also important to mention that previous studies of *S. cinnabarina* (cultivated material) do not describe this type of diterpenes.

Another labdane diterpenoid isolated from this species was identified as *trans*-communol (**4**), which was first obtained by reduction with LiAlH_4_ of the *trans*-communic acid methyl ester [16]. *Trans*-communol (**4**) has also been isolated from various natural sources, such as *Pinus thunbergii* Lamb. [37], *Chamaecyparis obtusa* (Siebold and Zucc.) Endl. [29], *C. formosensis* Matsum. [38] and *Fritillariae thunbergii* Miq. [39] among others. *Trans*-communol (**4**) has also been identified in the pyrolysis products of amber [40].

**Scheme 1:**
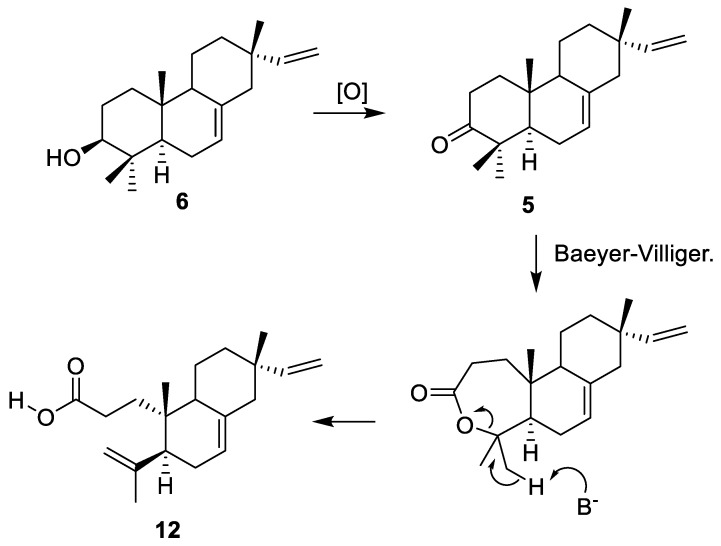
Biogenetic hypothesis to compound **12** starting from isopimarane **6**.

Two pimarane-type diterpenoids were also isolated from the dichloromethanic extract of this population of *S. cinnabarina* and identified as isopimara-7,15-dien-3-one (**5**) and isopimara-7,15-dien-3-ol (**6**). Their structures were established by spectroscopic means and comparison with literature data of previously described compounds isolated from other natural sources, including vegetable for example, *Guarea macrophylla* Vahl [41] and *Nepeta clarkei* Hook.f. [42], and even in the feces of the flying squirrel *Trogopterus xanthipes* Milne-Edwards [43]. According to the biogenetic hypothesis shown in Scheme 1, both isopimarane diterpenoids (**5** and **6**) can be considered precursors of 3,4-seco-isopimara-4(18),7,15-trien-3-oic acid (**12**), which was previously isolated from the foliar exudate of cultured *S. cinnabarina*.

Two sesquiterpenes **7** and **8** were also isolated from *S. cinnabarina* and identified based on their spectroscopic data as β-eudesmol (**7**) and cryptomeridiol (**8**) respectively. β-eudesmol (**7**) has been isolated from various plant sources, for example, from *Manglietia hookeri* Cubbil and W.W.Sm. (Magnoliaceae [44], *Ocimum basilicum* L. (Lamiaceae) [45] and *Salvia microphylla* Kunth (Lamiaceae) [46]. Cryptomeridiol (**8**) has been previously described from *Phaulopsis imbricata* (Forssk.) Sweet, *Artemisia pygmaea* A. Gray and *Blumea basalmifera* (L.) DC. [47].

The aromatic products **9**–**11**, also isolated from the dichloromethanic extract of *Salvia cinnabarina* were identified by spectroscopic means as the flavonoid salvigenin (**11**), phthalic acid (**9**) and a mixture of saturated fatty acids esters with tyrosol (4-hydroxyphenethyl alcohol) (**10**). Salvigenin (**11**) was originally isolated from *Salvia triloba L.*f [48] and has subsequently been described in several species of the genus, such as *S. barrelieri* Benth. [49], *S. dominica* L. [50], *S. apiana* Jeps. [51] and *S. sahendica* Boiss. and Bushe [52] among others. Several biological activities have been described for this flavonoid including anti-inflammatory, analgesic, anticancer and vasorelaxant [53].

Phthalic acid esters, derived from the esterification of phtalic acid (**9**) with long chain alcohols, have been isolated from various plant sources, for example, *Ajuga bracteosa* Wall. ex Benth. (Lamiaceae) [54], *Hedyotis uncinella* Hook. and Arn. (Rubiaceae) [55] and *Phyllantus rheedii* Wight. (Euphorbiaceae) [56] and also from marine organisms such as the red algae *Acantophora spicifera* (M. Vahl) Børgesen (Rhodomelaceae) [57]. Free phthalic acid is a product of the degradation of its esters by the action of some bacteria and it is known that it can have harmful effects by promoting the formation of reactive oxygen species causing cellular damage as described in *Malus prunifolia* (Willd.) Borkh [58]. Laboratory experiments determined that wheat, corn and soybean plants are capable of incorporating phthalic acid when the seeds are germinated in soil where this compound has been added [59]. Thus, the presence of **9** in *S. cinnabarina* raises the question of whether it is genetically part of the chemical composition, or whether this species incorporated phthalic acid from the soil where it grew.

The last aromatic compound was identified, based on its spectroscopic characteristics, as a mixture of esters of fatty acids with tyrosol. However, due to the low amount isolated, it was not possible to establish the size of the fatty acid chains. HPLC-MS analysis (Q-TOF) indicates the presence of a mixture of tyrosol fatty acids esters of more than 30 carbon atoms. These types of tyrosol derivatives have been previously isolated from different plant species [60,61] including a population of *Salvia microphylla* cultivated in Turkey [46]. Fatty acids esters of tyrosol had not been previously described in the chemical and biological analyses of Mexican sage.

### 2.1. Biological Activity

#### 2.1.1. Anti-Inflammatory Activity

Compounds 6β-hydroxy-*trans*-communic acid (**2**), *trans*-communic acid (**3**) and β-eudesmol (**7**) were tested as anti-inflammatory in the edema model induced by TPA. The percentages of inhibition obtained at 1.0 µmol/ear in a primary screening were 21.72%, 9.09% and 9.51%, respectively, so none of them showed significant activity.

#### 2.1.2. Inhibition of α-Glucosidase Activity

**Figure 7:**
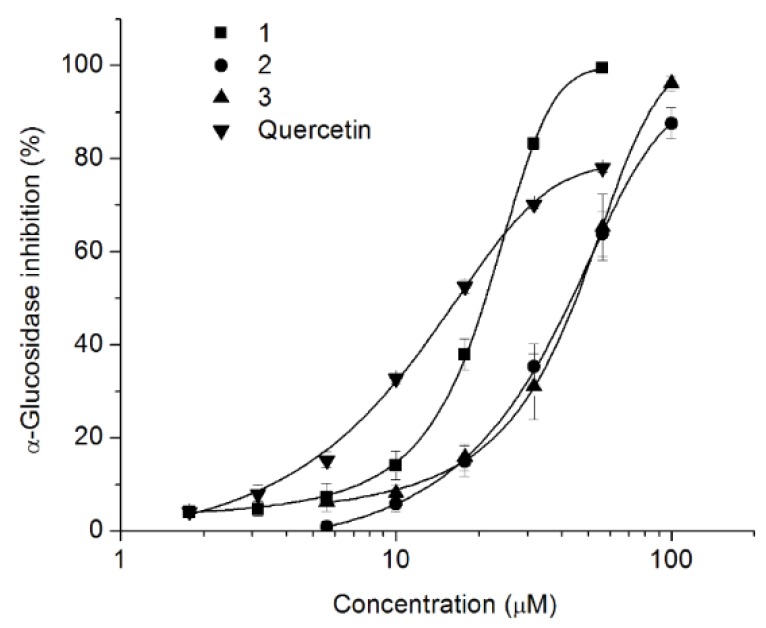
Concentration-response evaluation of compounds **1**–**3** isolated from *S. cinnabarina* on yeast α-glucosidase inhibition. Each value represents the mean of three independent experiments ± SEM; The data were analyzed by ANOVA followed by Dunnet post hoc test for comparison with control group.

Malonylcommunol (**1**), 6β-hydroxy-*trans*-communic acid (**2**) and *trans*-communic acid (**3**) were evaluated as α-glucosidase inhibitors in yeast and mammalian α-glucosidases. In a primary screening, using yeast α-glucosidase compounds **1**–**3** showed the highest inhibition of the enzyme, however the activity of the compounds decreased significantly in the mammalian α-glucosidase, evidencing a high specificity towards yeast α-glucosidase. Figure 7 shows that the effect of the evaluated compounds in yeast α-glucosidase is dependent of the concentration. Malonylcommunol (**1**) was the most active compound (IC_50_ 20.96 ± 0.58 µM), close to the reference compound quercetin (IC_50_ 16.72 ± 0.61 µM). Compounds **2** and **3** showed lower effect with IC_50_ = 43.74 ± 4.14 and 45.15 ± 3.76 respectively.

## 3. Materials and Methods

### 3.1. Experimental

The melting points (uncorrected) were determined on a Fisher-Johns (Fisher Scientific Company, Pittsburgh, PA, USA) apparatus. The optical rotations were measured on a Perkin-Elmer 323 polarimeter (Perkin Elmer Inc., London, UK). The ECD spectra were recorded on a Jasco J-1500 spectropolarimeter (Jasco, Tokyo, Japan) in MeOH at 1000 ppm. The IR spectra were obtained on a Bruker Tensor 27 spectrometer (Bruker, Ettlingen, Germany); 1D and 2D NMR experiments were performed on a Bruker Advance III HD spectrometer (Bruker BioSpin GmbH, Rheinstetten, Germany) at 500 MHz for ^1^H and 125 MHz for ^13^C and on a Bruker Advance III HD spectrometer (Bruker BioSpin GmbH, Rheinstetten, Germany) at 700 MHz for ^1^H and 175 MHz for ^13^C, using CDCl_3_ as solvent. Chemical shifts were referred to residual CHCl_3_ (*δ*_H_ = 7.26, *δ*_C_ = 77.16). The DART-MS data were obtained on a Jeol, The AccuTOF JMS-T100LC mass spectrometer. (Jeol Ltd., Tokyo, Japan) The X-ray data were collected on a Bruker D8 Venture (Bruker AXS GmbH, Karlsruhe, Germany) using CuKα (λ = 1.54178 Å). Silica gel 230-400 mesh (Macherey-Nagel, Nagel, Macherey Nagel, Düren, Germany), Sephadex LH-20 (Pharmacia Biotech AB, Uppsala, Sweden) and octadecyl-functionalized silica gel (Sigma-Aldrich, St. Louis, MO, USA) were used for column chromatography.

### 3.2. Plant Material

*Salvia cinnabarina* Martens and Galeotti was collected in Zoquitlan, State of Puebla, Mexico, in December 2017. Coordenates: 18°20′3.6″ N, 96°59′37.5″ W. Plant material was identified by Dr. Martha Martínez-Gordillo, and a voucher specimen (FCME 161531) was deposited at the Herbarium (FCME) of the Facultad de Ciencias (Mexico City, Mexico), UNAM. A photograph of the voucher is included as supplementary material.

### 3.3. Extraction and Isolation

The dried and powdered aerial parts of *S. cinnabarina* (370 g) were extracted exhaustively by percolation with CH_2_Cl_2_ (DCM). The DCM extract was concentrated at reduced pressure to yield 16 g of residue. The crude extract (16 g) was subjected to CC on silica gel using petrol: EtOAc (100: 0- 0: 100) as mobile phase to obtain 44 eluates, 250 mL each, which were combined in fifteen major fractions (A-O) after TLC evaluation. Fraction B (200 mg) was purified by CC on Sephadex LH-20, eluting with petrol: DCM: MeOH (3: 1: 1) as the mobile phase to obtain seventeen fractions (B1–B17), 10 mL each. Fraction B5 (25 mg) was purified by TLC using petrol: acetone (98: **2**) as mobile phase to give the compound isopimara-7,15-dien-3-one (**5**, 10.5 mg). *Trans*-communic acid (**3**, 146.2 mg) was isolated from fraction B7. A mixture of tyrosol derivatives (**10**, 7.2 mg) was identified in fraction C. Fraction D was subjected to CC on Sephadex LH-20 using MeOH as mobile phase to obtain fourteen fractions (D1-D14), 5 mL each. Fraction D10 was purified by TLC on ODS, using ACN: H_2_O (4: 1) to yield compounds isopimara-7,15-dien-3β-ol (**6**, 4.2 mg) and β-eudesmol (**7**, 10.3 mg). Fraction E (145 mg) was subjected to CC on Sephadex LH-20 (Pharmacia Biotech AB, Uppsala, Sweden) eluting with petrol: DCM: MeOH: Formic acid (3:1:1:0.5%) to obtain 54 fractions (E1–E54), 3 mL each. Fractions E24–E25 (25 mg) were combined and purified by CC using DCM: EtOH (98: 2) to obtain *trans*-communol (**4**, 4.7 mg). Phthalic Acid (**9**, 25 mg) was identified from fraction F. Fraction K (75 mg) was subjected to CC on Sephadex LH-20 using MeOH as mobile phase to obtain 15 fractions which were combined in five major fractions (KA-KE) after TLC evaluation. Fractions KB (70 mg) and KD (15 mg) were purified by TLC on ODS using MeOH: H_2_O (3: 1) to give malonylcommunol (**1**, 5.3 mg), and petrol: acetone (95: 5) to give salvigenin (**11**, 6 mg), respectively. Fraction L (938 mg) was subjected to CC on Sephadex LH-20 using MeOH as mobile phase to obtain fifteen fractions (L1–L15), 20 mL each, which were combined in four mayor fractions (LA-LD) after TLC evaluation. Fraction LC (302 mg) was subjected to CC on Sephadex LH-20 using petrol: DCM: MeOH (1: 1: 3) to obtain 20 fractions, 5 mL each, which were combined in seven major fractions (LCA-LCG); Fraction LCD afforded cryptomeridiol (**8**, 9.3 mg), while 6β-hydroxy-*trans*-communic acid (**2**, 40.3 mg) was isolated from fraction LD.

*Malonylcommunol* (**1**): white powder; m.p. 85–90 °C; [α]_D_^589^ = +2.2 (*c* 0.10, MeOH); IR (CHCl_3_) ν_max_ 3607, 3512, 2933, 2854, 1732, 1624 cm^−1^; ^1^H and ^13^C NMR, see Table 1; HRDARTMS *m*/*z* [M + H]^+^ 375.25347 (calculated for C_23_H_35_O_4_, 375.25353).

*6*β*-Hydroxy-trans-communic acid* (**2**): white powder; m.p. 170–173 °C; [α]_D_^589^ = +23.0 (*c* 0.50, MeOH); IR (CHCl_3_) ν_max_ 3601, 3591, 2941, 2853, 1726, 1519, 1406, 1239, 1223 cm^−1^; ^1^H and ^13^C NMR, see Table 2; HRDARTMS *m*/*z* [M + H]^+^ 319.24747 (calculated for C_20_H_31_O_3_, 319.24845).

*Trans*-communic acid (**3**): colorless crystals; m.p. 118–120 (reported 135–137 °C); [α]_D_^589^ = +45.3 (*c* 0.40, CHCl_3_) (reported + 47.7; *c* 0.45, CHCl_3_); NMR data were essentially the same as reported [29].

*Trans communol* (**4**): colorless oil; [α]_D_^589^ = +4.6 (*c* 0.61, CHCl_3_) ((reported + 18.0, CHCl_3_); IR (CHCl_3_) ν_max_ 3629, 2872, 2852, 1723, 1642, 1451, 1384, 1209, 1018, 908, 895 cm^−1^. ^1^H NMR (CDCl_3_, 500 MHz) *δ* 6.33 (1H, dd, *J* = 17.4, 10.7 Hz, H-14), 5.40 (1H, t, *J* = 6.5 Hz, H-12), 5.04 (1H, d, *J* = 17.4 Hz, H-15a), 4.88 (1H, d, *J* = 10.7 Hz, H-15b), 4.81 (1H, brq, *J* = 1.5 Hz, H17a), 4.46 (1H, brq, *J* = 1.5 Hz, H17b), 3.77 (1H, d, *J* = 10.8 Hz, H19a), 3.41 (1H, d, *J* = 10.8 Hz, H19b), 2.42–2.37 (2H, m, H-7a, H-11a), 2.13 (1H, ddd, *J* = 16.5, 11.0, 6.8 Hz, H-11b), 1.96 (1H, td, *J* = 12.8, 6.7 Hz, H-7b), 1.77–1.86 (4H, m, H-1a, H-3a, H-6a, H-9), 1.75 (3H, brs, CH_3_-16), 1.52 (2H, m, CH_2_-2), 1.32 (1H, td, *J* = 12.6, 4.1 Hz, H-6b), 1.28 (1H, m, H-5), 1.13 (1H, td, *J* = 12.3, 5.4 Hz, H1b), 1.01-1.95 (1H, m, H-3b), 0.99 (3H, s, CH_3_-18), 0.70 (3H, s, CH_3_-20); ^13^C NMR (CDCl_3_, 126 MHz) *δ* 148.2 (C-8), 141.8 (C-14), 134.1 (C-12), 133.6 (C-13), 110.0 (C-17), 107.9 (C-15), 65.3 (C-19), 57.3 (C-9), 56.4 (C-5), 39.6(C-10), 39.3 (C-1), 39.0 (C-4), 38.5 (C-7), 35.5 (C-3), 27.2 (C-18), 24.4 C-6), 23.4 (C-11), 19.2 (C-2), 15.4 (C-20), 12.0 (C-16). DARTMS *m*/*z* 289.

*Isopimara-7,15-dien-3-one* (**5**): white powder; m.p. 80–82 °C (reported 91–92 °C); [α]_D_^589^ = −70 (*c* 0.26, CHCl_3_) ((reported-82, CHCl_3_, c 2.0) [62]; IR (CHCl_3_) ν_max_ 2919, 2869, 1702, 1638, 1455, 1386, 1206, 1000, 915, 845 cm^−1^. ^1^H NMR (CDCl_3_, 700 MHz) *δ* 5.81 (1H, dd, *J* = 17.5, 10.7 Hz, H-15), 5.41(1H, brs, H-7), 4.94 (1H, dd, *J* = 17.5, 1.3 Hz, H-16a), 4.88 (1H, dd, *J* = 10.7, 1.3, H-16b), 2.70 (1H, td, *J* = 14.6, 5.3 Hz, H-2a), 2.70 (1H, td, *J* = 14.6, 3.8 Hz, H-2b), 2.13–2.08 (2H, m, H-1, H-6), 1.98 (1H, brd, *J* = 13.9 Hz, H-14a), 1.94 (1H, td, *J* = 13.9, 2.5 Hz, H14b), 1.90 (1H, m, H-6b), 1.71 (1H, m, H-9), 1.60 (2H, m, H-11), 1.55 (1H, dd, *J* = 11.9, 4.1 Hz, H-5), 1.52–1.49 (2H, m, H1b, H12a), 1.35–1.45 (2H, m, H-11b, H-12b), 1.13 (3H, s, CH3-19), 1.09 (3H, s, CH3-20), 1.07 (3H, s, CH3-18), 0.89 (3H, s, CH3-17); ^13^C NMR (CDCl_3_, 175 MHz) *δ* 217.1 (C-3), 150.2 (C-15), 135.8 (C-8), 121.3 (C-7), 109.6 (C-16), 51.9 (C-5), 51.2 (C-9), 47.6 (C-4), 46.1 (C-14), 38.3 (C-1), 37.0 (C-13), 36.2 (C-152), 35.4 (C-10), 34.9 (C-2), 25.7 (C-18), 24.0 (C-6), 22.8 (C-19), 21.7 (C-17), 20.4 (C-11), 15.0 (C-20).

*Isopimara-7,15-dien-3-ol* (**6**): colorless oil; [α]_D_^589^ = −8.0 (*c* 0.18, CHCl_3_; NMR data were essentially the same as reported.

### 3.4. Computational Details

3D models for compounds **3** and **4** were built, and geometry optimized using Spartan′10 utilizing a Merck Molecular Force Field (MMFF). Conformational analysis was performed with the same software under a PM3 semiempirical force field. The resulting conformers were filtered and checked for redundancy. All conformers within 4 kcal/mol were minimized, optimized and the thermochemical properties, IR and vibrational frequencies calculated using the DFT-B3LYP/DGDZVP force field in Gaussian 09. The TD-SCF with the default solvent model was used to perform the ECD calculations of the major conformers in MeOH solution at the B3LYP/6-31G* (d) level of theory. The calculated excitation energy (nm) and rotatory strength (*R*) in dipole velocity (*R_vel_*) form were simulated into an ECD curve using the Harada–Nakanishi equation (Equation (1)) as implemented in the SpecDis software [63,64,65]. All calculations were performed on the HP Cluster Platform 3000SL “Miztli.”

(1) $$\Delta \varepsilon \left(\upsilon \right)=\overset{n}{\underset{i=1}{\sum }}\;\Delta \varepsilon_{i}\left(\upsilon \right)=\overset{n}{\underset{i=1}{\sum }}=\;\frac{R_{i}}{2.296\;\times \;10^{-39}\sqrt{\pi }}\frac{\upsilon_{i}}{\sigma }e\left[-{\left(\frac{\upsilon -\upsilon_{i}}{\sigma }\right)}^{2}\;\right]$$

### 3.5. Single-Crystal X-ray Diffraction Analysis for Trans-Communic Acid (***3***)

A colorless crystal was selected for experimental diffraction and mounted in a D8 venture Κ geometry diffractometer (Bruker AXS GmbH, Karlsruhe, Germany) with micro-focus X-ray source Cu kα radiation (λ = 1.54178 Å). The detector was placed at 50 mm from the crystal. Frames were collected with a scan width of 0.3° in the ω scan and the exposure time of 10 sec/frame at 298 K. Frames were integrated with the Bruker SAINT software package (Bruker AXS Inc., Madison, Wisconsin, USA) using a narrow-frame integration algorithm. Systematic absences and intensity statistics were used in system orthorhombic, space group P2_1_2_1_2_1_. The structure was solved using direct methods using SHELXS-2014/7 program [66]. Hydrogen atoms were input at calculated positions and allowed to ride on the atoms to which they are attached. Thermal parameters were refined for hydrogen atoms on the aromatic ring and methylene using a *Ueq* = 1.2 Å and a *Ueq* = 1.5 Å for methyl groups to precedent atom in all cases. The final cycle of refinement was carried out on all non-zero data using SHELXL-2014/7 [66]. The Flack parameter was determined as 0.1(1) and confirmed with Bayesian parameters [67] P2 (true) = 1.000, P3 (false) = 0.3E-2 and Pearson z= 0.38(11).

The *trans*-communic acid (**3**) was determined with two molecules crystallographically independent with an labdane-type structure, in according with Cremer and Pople puckering parameters [68] A-B rings has a chair conformation (A1 ring Q = 0.540(3), Theta = 0.0(3)°, Phi = 100(19)°, B1 ring Q = 0.576(3), Theta = 0.6(3) Deg, Phi = 289(6)°, A2 ring (Q) = 0.542(3), Theta = 3.4(3)°, Phi = 125(6)° and B2 ring (Q) = 0.593(3), Theta = 2.0(3)°, Phi = 263(6)°). The carboxylic acid is in an axial orientation in both molecules, and is forming a H-bonding with other carboxylic acid by symmetry code 1 − x, 1/2 + y, 1/2 − z.

Crystallographic data (excluding structure factors) were deposited at the Cambridge Crystallographic Data Centre (CCDC) under the reference numbers CCDC 1983658, and copies of the data can be obtained free of charge upon application to the CCDC, 12 Union Road, Cambridge CB2 IEZ, UK. Fax: +44-(0)1223-336033 or e-mail: deposit@ccdc.cam.ac.uk.

### 3.6. TPA-Induced Edema Model

Animals. Male CD-1 mice weighing 25–30 g were maintained under standard laboratory conditions in the animal house (temperature 24 ± 2 °C) in a 12/12 h light−dark cycle, being fed laboratory diet and water ad libitum, following the Mexican official norm NOM-062-Z00-1999. The experimental procedures were approved by Internal Ethic Committee (CICUAL-IQ-004-17).

The TPA-induced ear edema assay in mice was performed as reported [69]. A solution of TPA (2.5 µg) in ethanol (10 µL) was applied topically to both faces (5 µL each ear) of the right ear of the mice, after 10 min the solutions of the test substances in their respective solvents were applied (10 µL each face). The left ear received ethanol (10 µL) then 20 µL of the respective solvent. The mice were killed with CO_2_ four hours later. A 7 mm diameter plug was removed from each ear. The swelling was assessed as the difference in weight between the left and the right ear. Control animals received the correspondent solvent in each case. Edema inhibition (EI%) was calculated by the equation EI% = 100 − (B × 100/A), where A is the edema induced by TPA alone and B is the edema induced by TPA plus sample. Indomethacin and celecoxib were used as reference compounds.

### 3.7. Inhibition of α-Glucosidase

#### 3.7.1. Inhibition of Yeast α-Glucosidase

α-Glucosidase inhibition was evaluated using an adapted method of Xiao-Ping et al. 2010 and Zhou et al. 2010 [70,71]. A solution of tested samples (25 µL) in DMSO-H_2_O 1:1 was added to 150 µL of phosphate buffer solution (PBS, 67 mM, pH 6.8) and incubated at 37 °C for 10 min with 25 µL of reduced glutation (3 mM in PBS) and 25 µL of 0.2 U mL^−1^ in PBS solution of α-glucosidase type I (Sigma cat. G5003-100UN). The substrate solution (25 µL, 23.2 mM *p*-nitrophenyl-α-D-glucopyranoside, Sigma N1377-1G, in PBS) was added and incubated at 37 °C for an additional 15 min and shaken. Reaction mixture was stopped with CaCO_3_ 1M (50 µL) and after 5 min agitation the optical density was determined at 405 nm. Quercetin was used as positive control. The inhibition percentage was calculated by the equation: Inhibition (%) = [(Acontrol − Asample) / Acontrol] × 100. Where A is the absorbance at 405 nm of sample and control.

#### 3.7.2. Mammalian α-Glucosidadse Inhibition Assay

Mammalian α-glucosidase was prepared following the modified method of Jo [72]. Rat-intestinal acetone powder (100 mg) was rehydrated with 4 mL of 67 mM ice cold phosphate buffer (pH 6.8). After homogenized in an OMNI International Tissue Homogenizer (Omni International, Inc., Kennesaw, GA, USA) (125 model) for 3 min at 4 °C, the suspension was centrifuged (13,400 rcf, 4 °C, 30 min) and the resulting supernatant was used for the assay. A reaction mixture containing 175 µL phosphate buffer (67 mM, pH 6.8), 25 µL of α-glucosidase supernatant and 25 µL of sample at different concentrations (dissolved in DMSO 50%), was pre-incubated for 10 min at 37 °C. Then 25 µL of 23.2 mM PNP-G was added as a substrate. After further incubation of 15 min at 37 °C, the reaction was stopped whit 50 µL of Na_2_CO_3_ (1 M). Acarbose and miglitol were used as a positive control and DMSO 5% as negative control. Enzyme activity was quantified by measuring the absorbance at 405 nm in a BioTek microplate reader Synergy HT (BioTek Instruments, Winooski, VT. USA). Experiments were done in triplicates. The percentage of enzyme inhibition by the sample was calculated by the following formula: % Inhibition = [(AC − AS) / AC)] × 100, where AC is the absorbance of the negative control and AS is the absorbance of the tested sample. The concentration of an inhibitor required to inhibit 50% of enzyme activity under the mentioned assay conditions is defined as the inhibition concentration 50 (IC_50_)

## 4. Conclusions

From the dichloromethane extract of a wild population of *Salvia cinnabarina*, several natural products were isolated, including two unpublished labdane-type diterpenoids named malonylcommunol (**1**) and 6β-hydroxy-*trans*-communic acid (**2**). Two already known labdane diterpenoids, *trans*-communic acid (**3**) and *trans*-communol (**4**) were also isolated and identified by spectroscopic means and comparison with literature date. Two isopimarane-type diterpenoids **5** and **6** were isolated together with two eudesmane-type sesquiterpenoids identified as β-eudesmol (**7**) and cryptomeridiol (**8**). Three aromatic natural products identified as phtalic acid (**9**), tyrosol derivatives (**10**) and the flavone salvigenin (**11**) were also isolated from this plant. Compounds **5** and **6** could be considered as biogenetic precursor of compound **12**, a bioactive *seco*-*iso*pimarane diterpenoid previously isolated from a cultivated population of *S. cinnabarina*. This work represented the first phytochemical analysis of a wild population of this plant.

Some products were tested in the TPA induced edema, anti-inflammatory assay with no significant results. The assay of compounds **1**–**3** as α-glucosidase inhibitors indicated high specificity towards yeast α-glucosidase. Malonylcommunol (**1**) was the most active compound (IC_50_ 20.96 ± 0.58 µM), near to the reference compound quercetin.

## Figures and Tables

**Table 1:** NMR Data (^1^H 700 MHz and ^13^C 175 MHz, CDCl_3_) of **1**.

Position	δ_C_	Type ^a^	*δ*_H_ (*J* In Hz)	HMBC
1a	39.0	CH_2_	1.83, brd (12.5)	2, 3, 5, 20
1b			1.13, td (12.5, 5.4)	2, 3, 5, 10, 20
2	19.0	CH_2_	1.52, m	1, 3, 4
3a	36.2	CH_2_	1.73, brd (12.8)	1, 2, 4, 5, 18, 19
3b			1.04, td (12.8, 5.3)	1, 2, 4, 18, 19
4	37.6	C		
5	56.2	CH	1.30, brd (12.5)	1, 6, 7, 18, 19, 20
6a	24.3	CH_2_	1.83, m	5, 8
6b			1.34, dq (12.5, 3.9)	5, 7
7a	38.3	CH_2_	2.39, m	5, 6, 9, 17,
7b			1.97, td (12.5, 4.6)	5, 6, 8, 17
8	147.7	C		
9	57.2	CH	1.75, brd (13.0)	8, 11, 12, 17, 20
10	39.5	C		
11a	23.3	CH_2_	2.36, m	8, 9, 12, 13, 14,* 17 *
11b			2.14, m	8, 9, 12, 13, 14 *
12	133.8	CH	5.39, brt (6.2)	9, 11, 14, 16
13	133.7	C		
14	141.7	CH	6.32, dd (17.4, 10.7)	12, 13, 16
15a	110.1	CH_2_	5.04, d (17.4)	12,* 13, 14
15b			4.88, d (10.7)	12,* 13, 14
16	12.0	CH_3_	1.74, s	12, 13, 14
17a	108.2	CH_2_	4.82, brs	6,* 7, 8
17b			4.47, brs	6,* 7, 8, 9
18	27.6	CH_3_	0.97, s	3, 5, 19,
19a	68.5	CH_2_	4.38, d (10.9)	3, 5, 3′, 18
19b			3.95, d (10.9)	3, 5, 3′, 18
20	15.4	CH_3_	0.72, s	1, 5, 9, 10,
1′	169.6	C		
2′	40.7	CH_2_	3.41, brs	1′, 3′, 19
3′	168.3	C		

^a^ According to HSQC spectrum; * Through four-bonds interaction.

**Table 2:** NMR Data (^1^H 700 MHz and ^13^C 175 MHz, CDCl_3_) of **2**.

Position	δ_C_	Type ^a^	δ_H_ (*J* In Hz)	HMBC
1a	20.2	CH_2_	1.76, qd (14.3, 3.5)	3
1b			1.56, dt (14.3, 2.8)	10
2a	41.6	CH_2_	1.88, brd (13.0)	1, 3, 10, 20 *
2b			1.19, td (13.0, 3.7)	1, 3, 9, 10, 20 *
3a	40.4	CH_2_	2.38, brd (13.0)	1, 2, 4
3b			1.01, td (13.0, 3.7)	1, 4, 5, 18, 19
4	46.6	C		
5	57.5	CH	1.46, brs	4, 6, 9, 10, 18, 19, 20
6	67.9	CH	4.52, brq (2.6)	4, 5, 7, 8, 10
7a	45.4	CH_2_	2.50, dd (13.6, 2.6)	5, 6, 8, 9, 17
7b			2.34, brd (13.6)	6, 8, 9, 17
8	133.9	C		
9	56.7	CH	1.82, brd (11.1)	5, 7, 8, 10, 11, 17, 20
10	41.3	C		
11a	23.4	CH_2_	2.40, m	8, 9, 12, 13, 15
11b			2.23, ddd (16.6, 11.3, 6.4)	9, 12, 13
12	132.9	CH	5.41, brt (6.4)	9, 11, 14, 16
13	142.3	C		
14	141.4	CH	6.32, dd (17.4, 10.8)	12, 16
15a	110.3	CH_2_	5.06, d (17.4)	12, 14
15b			4.90, d (10.8)	12, 14
16	11.9	CH_3_	1.76, s	12, 14
17a	111.9	CH_2_	5.03, brs	7, 8, 9
17b			4.76, brs	7, 8, 9
18	28.4	CH_3_	1.33, s	3, 4, 5
19	180.2	C		
20	15.8	CH_3_	0.87, s	9, 10

^a^ According to HSQC spectrum; * Through four-bonds interaction.

## Supplementary Materials

The following are available online, Figure S1: ^1^H NMR (CDCl_3_, 700 MHz) spectrum of **1**, Figure S2. ^1^H NMR (CDCl_3_ + D_2_O, 700 MHz) spectrum of 1, Figure S3: ^13^C NMR (CDCl_3_, 175 MHz) spectrum of **1**, Figure S4: COSY NMR (CDCl_3_, 700 MHz) spectrum of **1**, Figure S5: HMBC NMR (CDCl_3_, 700 MHz) spectrum of **1**, Figure S6: HSQC NMR (CDCl_3_, 700 MHz) spectrum of **1**, Figure S7: NOESY NMR (CDCl_3_, 700 MHz) spectrum of **1**, Figure S8: ^1^H NMR (CDCl_3_, 700 MHz) spectrum of **2**, Figure S9: ^13^C NMR (CDCl_3_, 175 MHz) spectrum of **2**, Figure S10: COSY NMR (CDCl_3_, 700 MHz) spectrum of **2**, Figure S11: HMBC NMR (CDCl_3_, 700 MHz) spectrum of **2**, Figure S12: HSQC NMR (CDCl_3_, 700 MHz) spectrum of **2**, Figure S13: NOESY NMR (CDCl_3_, 700 MHz) spectrum of **2**. Figure S14. ^1^H NMR (CDCl_3_, 700 MHz) spectrum of **4**, Figure S15. ^13^C NMR (CDCl_3_, 175 MHz) spectrum of **4**, Figure S16. ^1^H NMR (CDCl_3_, 700 MHz) spectrum of **5**, Figure S17. ^13^C NMR (CDCl_3_, 175 MHz) spectrum of **5**, Figure S18. Herbarium specimen of *Salvia cinnabarina* and Table S1. Primary screening of the Inhibitory effect of compounds **2**, **3** and **7** on TPA-induced inflammation in a mouse model, Table S2. Primary screening of inhibition of mammalian α-glucosidase activity for compounds **1** and **2**.
