# Supplementary material for: Structural Elucidation of Malonylcommunol and 6β-Hydroxy-trans-communic Acid, Two Undescribed Diterpenes from Salvia cinnabarina. First Examples of Labdane Diterpenoids from a Mexican Salvia Species
Source: Molecules. 2020 Apr 15;25(8):1808. doi: 10.3390/molecules25081808 (PMC7222005; doi:10.3390/molecules25081808)
Supplement: Supplementary file 1 [file molecules-25-01808-s001.pdf]

Article

Structural elucidation of malonylcommunol and 6*β*-hydroxy-*trans*-communic acid, two undescribed diterpenes from *Salvia cinnabarina*. First examples of labdane diterpenoids from a Mexican *Salvia* species

Celia Bustos-Brito [1], Antonio Nieto-Camacho [1], Simón Hernandez-Ortega [1], José Rivera-Chávez [1], Leovigildo Quijano [1,*], and Baldomero Esquivel [1,*]

[1] Instituto de Química, Universidad Nacional Autónoma de México, Circuito Exterior, Ciudad Universitaria, Mexico City., 04510 México; celia.bustos@iquimica.unam.mx (C.B.-B); anieto@unam.mx (A.N), simonho@unam.mx (S.H.-O); jrivera@iquimica.unam.mx (J. R.-C.); quijano@unam.mx (Q.L.); baldo@unam.mx (B.E.)

* Correspondence: quijano@unam.mx; Tel.:+52-55-5622-4411 (Q.L.); baldo@unam.mx; Tel.:+52-55-5622-4448 (B.E.)

Supporting Information

Table of Contents

Figure S1. ^{1}H NMR ($CDCl_3$, 700 MHz) spectrum of **1**
Figure S2. ^{1}H NMR ($CDCl_3$ + D_2O, 700 MHz) spectrum of **1**
Figure S3. ^{13}C NMR ($CDCl_3$, 175 MHz) spectrum of **1**
Figure S4. COSY NMR ($CDCl_3$, 700 MHz) spectrum of **1**
Figure S5. HMBC NMR ($CDCl_3$, 700 MHz) spectrum of **1**
Figure S6. HSQC NMR ($CDCl_3$, 700 MHz) spectrum of **1**
Figure S7. NOESY NMR ($CDCl_3$, 700 MHz) spectrum of **1**
Figure S8. ^{1}H NMR ($CDCl_3$, 700 MHz) spectrum of **2**
Figure S9. ^{13}C NMR ($CDCl_3$, 175 MHz) spectrum of **2**
Figure S10. COSY NMR ($CDCl_3$, 700 MHz) spectrum of **2**
Figure S11. HMBC NMR ($CDCl_3$, 700 MHz) spectrum of **2**
Figure S12. HSQC NMR ($CDCl_3$, 700 MHz) spectrum of **2**
Figure S13. NOESY NMR ($CDCl_3$, 700 MHz) spectrum of **2**
Figure S14. ^{1}H NMR (CDCl3, 700 MHz) spectrum of **4**
Figure S15. ^{13}C NMR (CDCl3, 175 MHz) spectrum of **4**
Figure S16. ^{1}H NMR (CDCl3, 700 MHz) spectrum of **5**
Figure S17. ^{13}C NMR (CDCl3, 175 MHz) spectrum of **5**
Figure S18. Herbarium specimen of *Salvia cinnabarina* collected by Dr. Baldomero Esquivel-Rodríguez collected in Zoquitlan, State of Puebla, Mexico, in December 2017.
Table S1. Primary screening of the Inhibitory effect of compounds 2, 3 and 7 on TPA-induced inflammation in a mouse model.
Table S2. Primary screening of inhibition of mammalian α-glucosidase activity for compounds **1** and **2**.

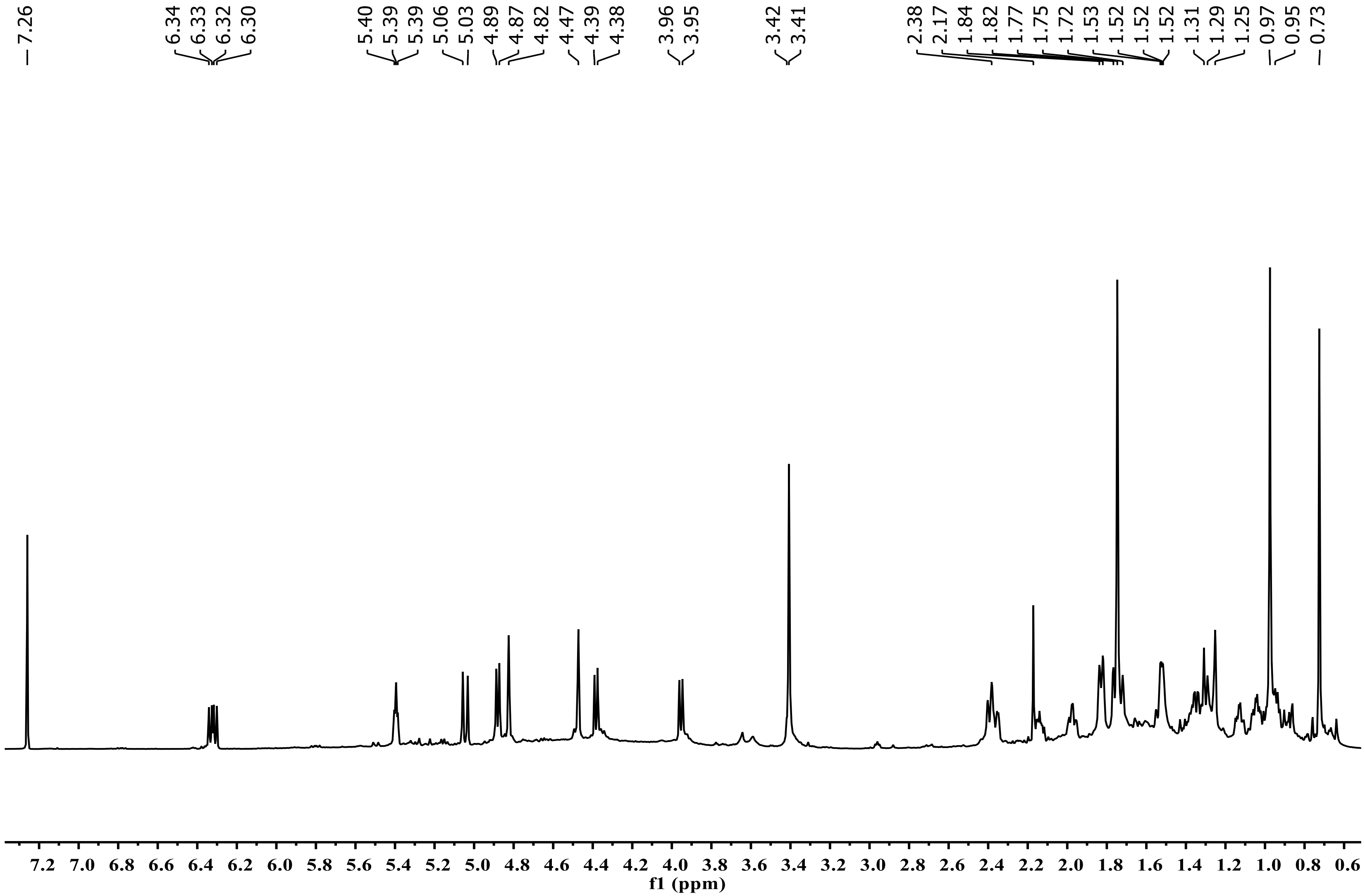

Figure S1. ^{1}H NMR ($CDCl_3$, 700 MHz) spectrum of **1**

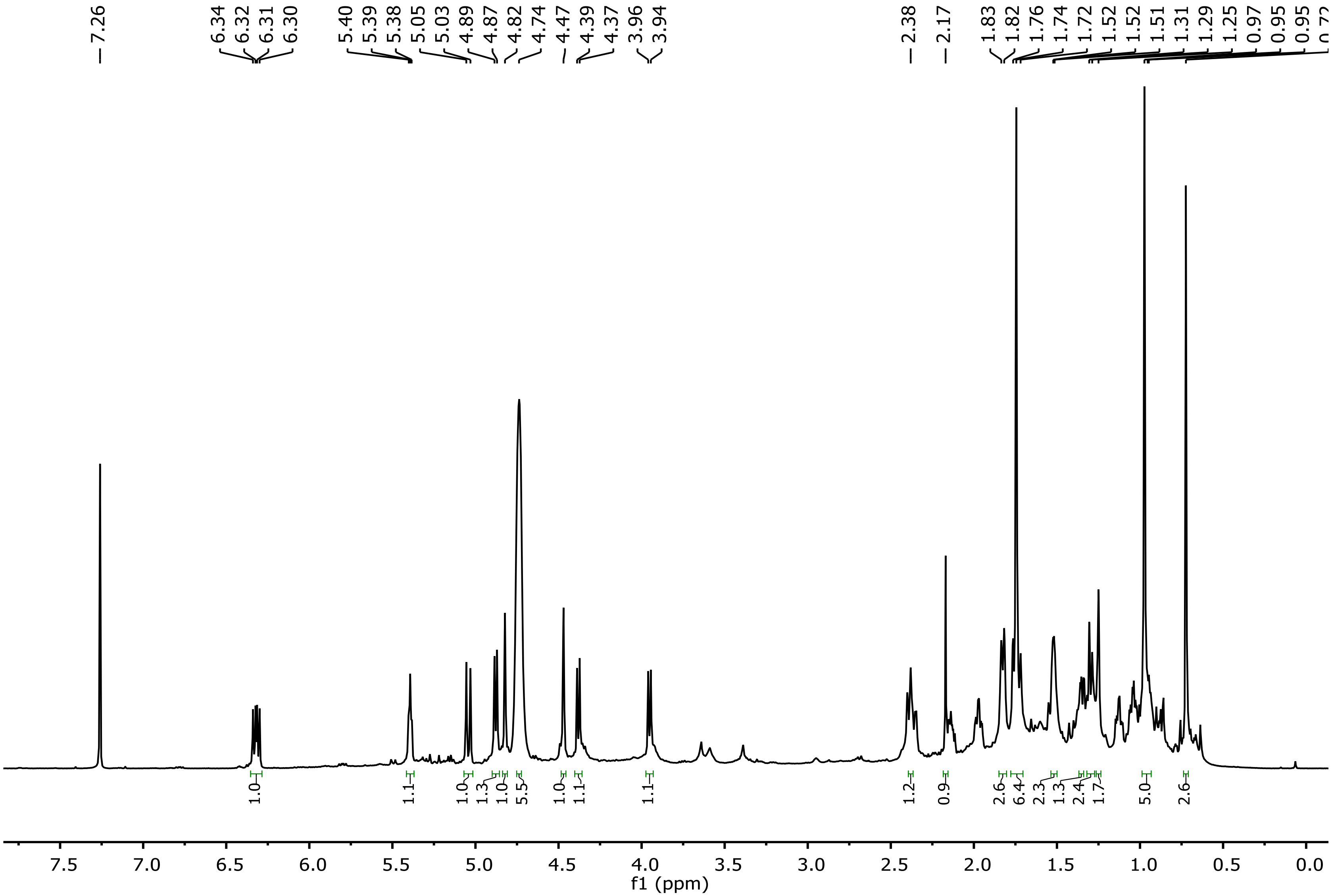

Figure S2. ^{1}H NMR ($CDCl_3 + D_2O$, 700 MHz) spectrum of **1**

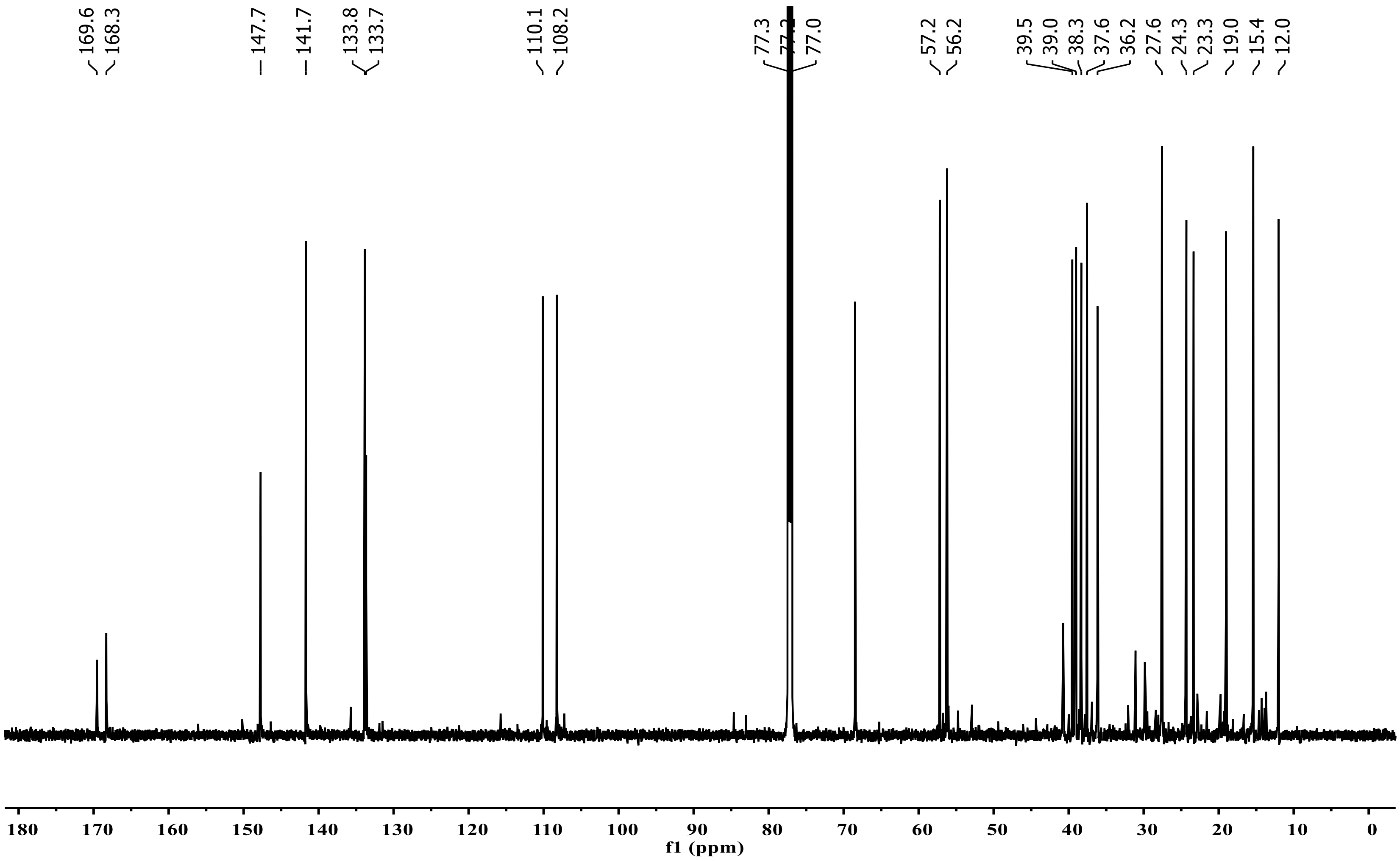

Figure S3. ^{13}C NMR ($CDCl_3$, 175 MHz) spectrum of **1**

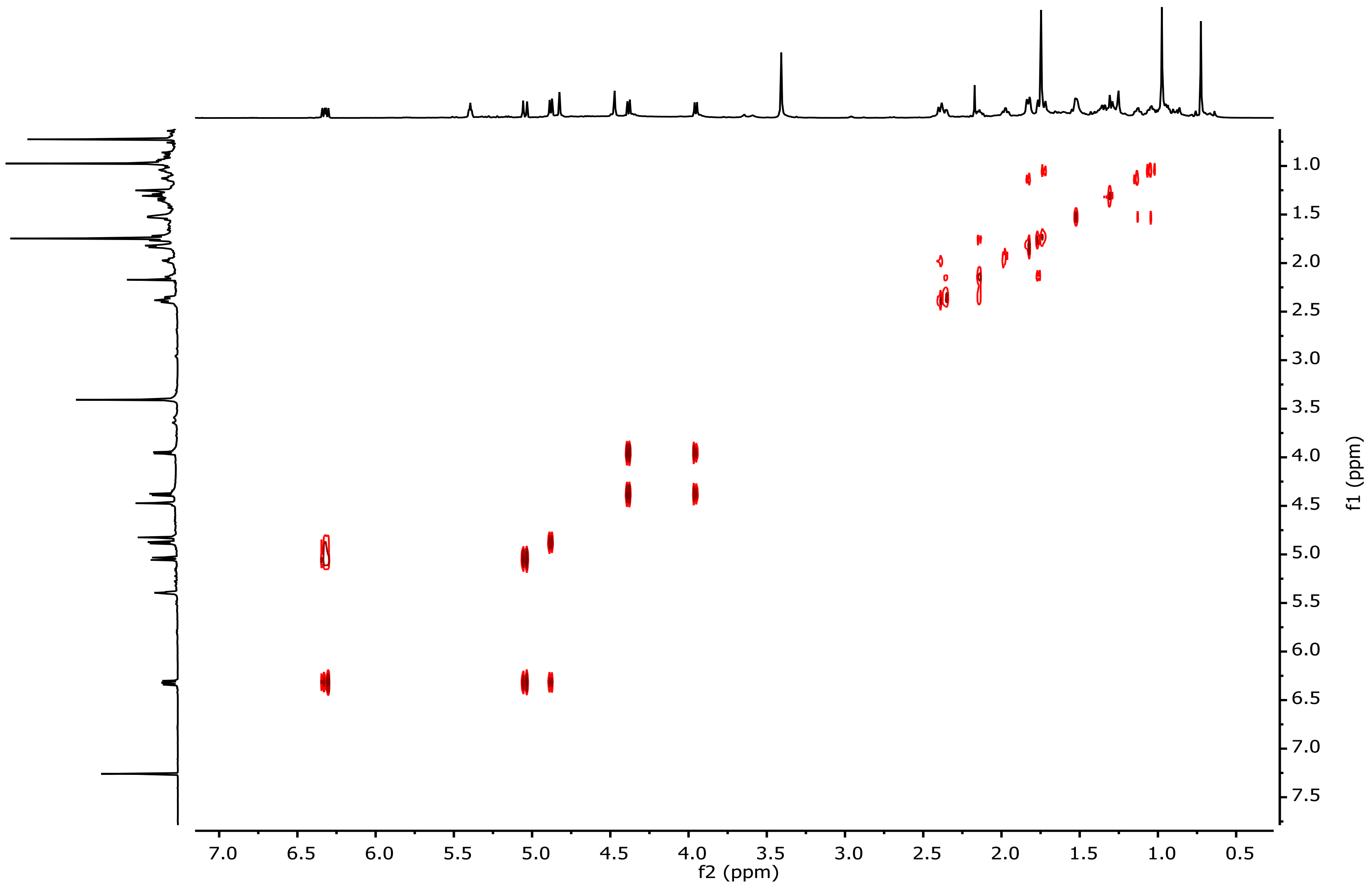

Figure S4. COSY NMR ($CDCl_3$, 700 MHz) spectrum of **1**

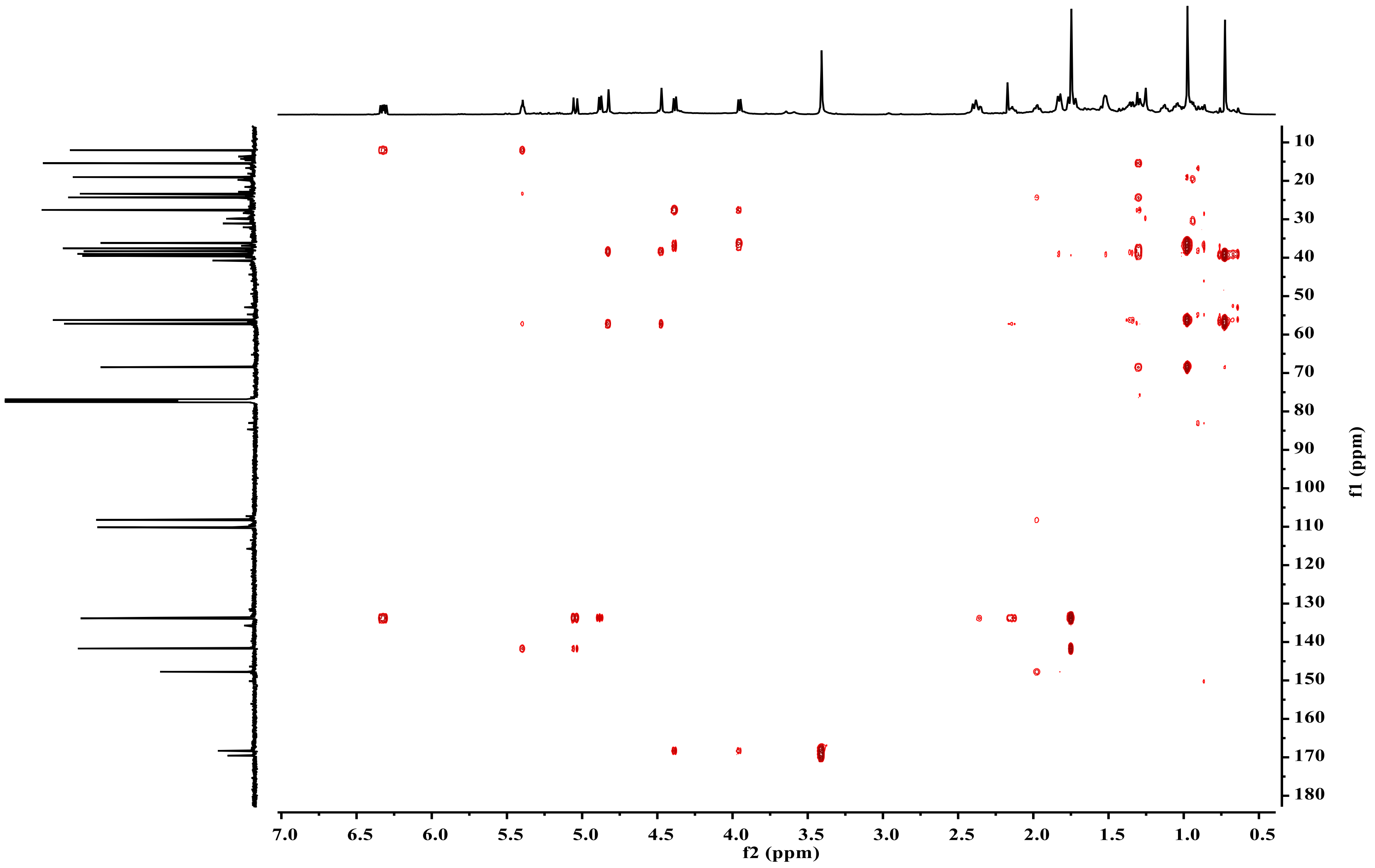

Figure S5. HMBC NMR ($CDCl_3$, 700 MHz) spectrum of **1**.

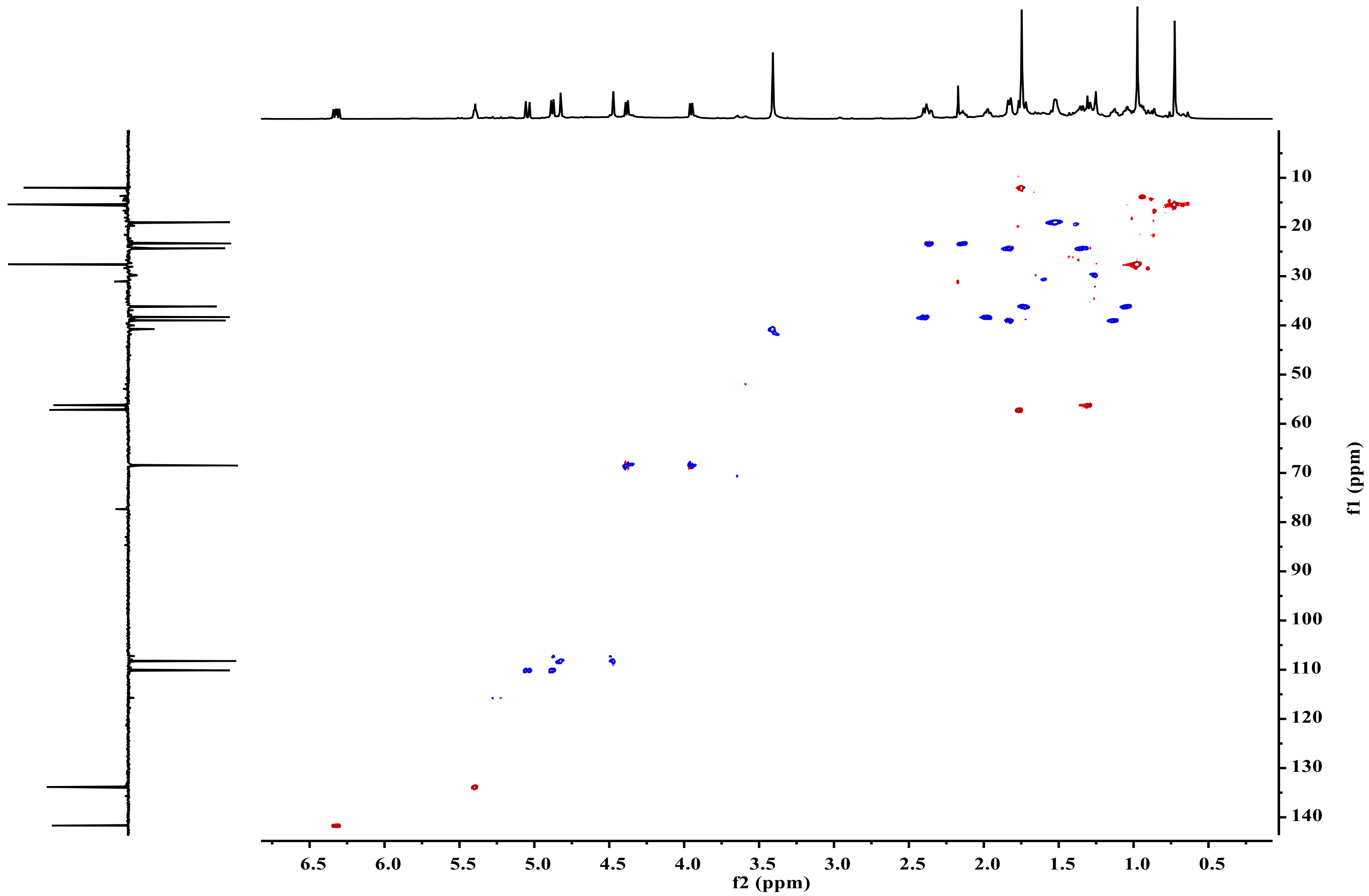

Figure S6. HSQC NMR ($CDCl_3$, 700 MHz) spectrum of **1**

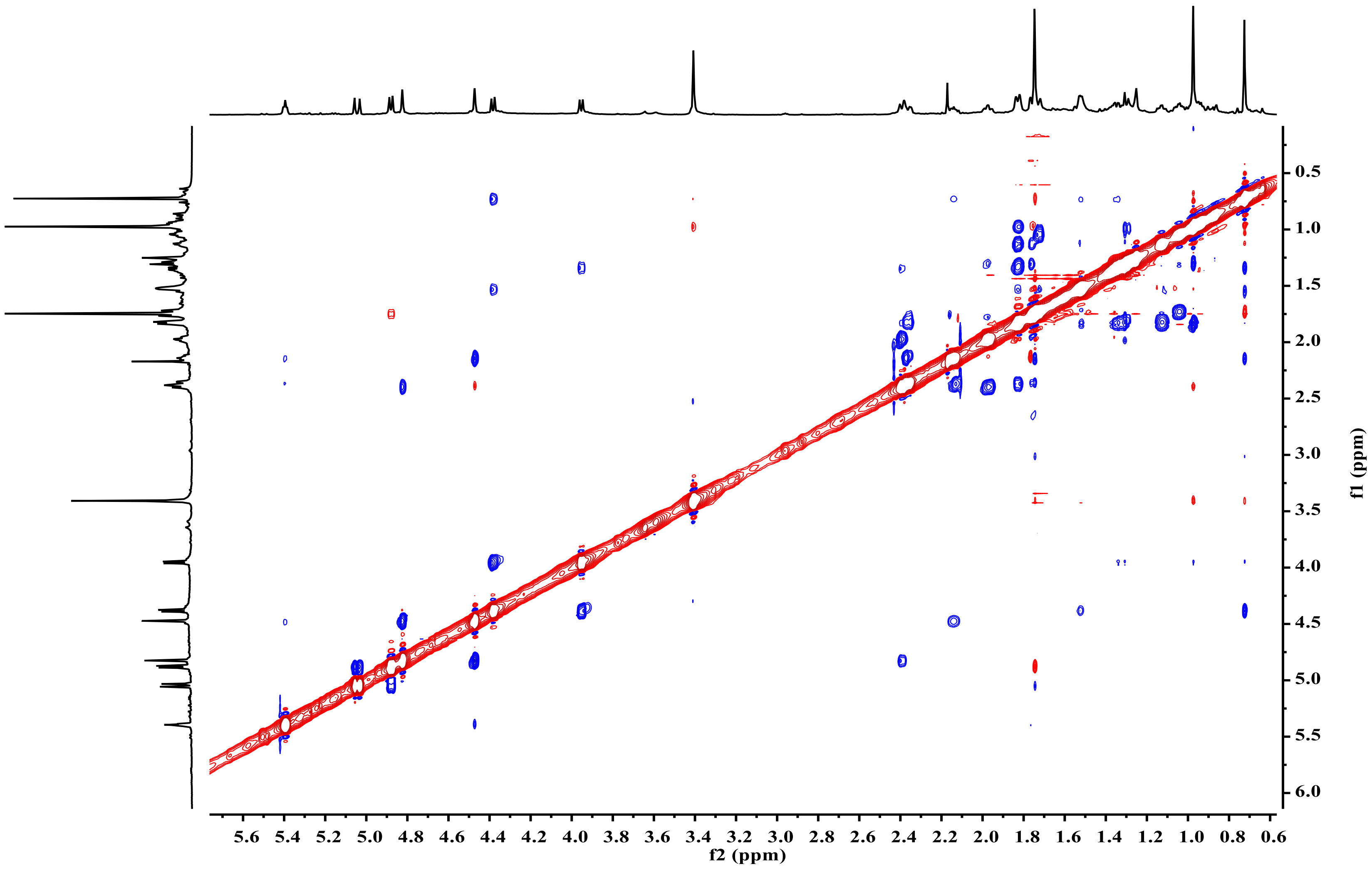

Figure S7. NOESY NMR ($CDCl_3$, 700 MHz) spectrum of **1**

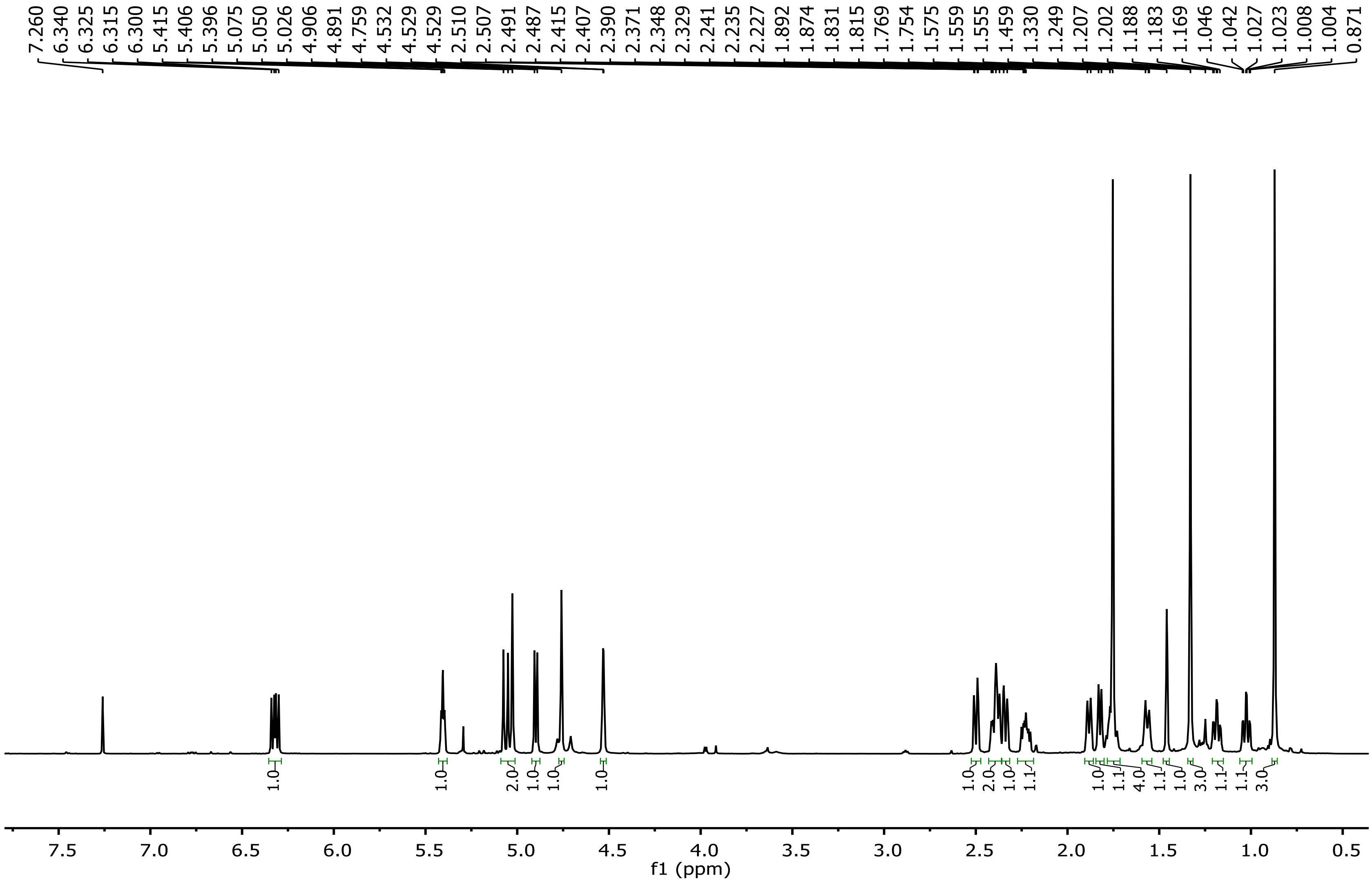

Figure S8. ^{1}H NMR ($CDCl_3$, 700 MHz) spectrum of **2**

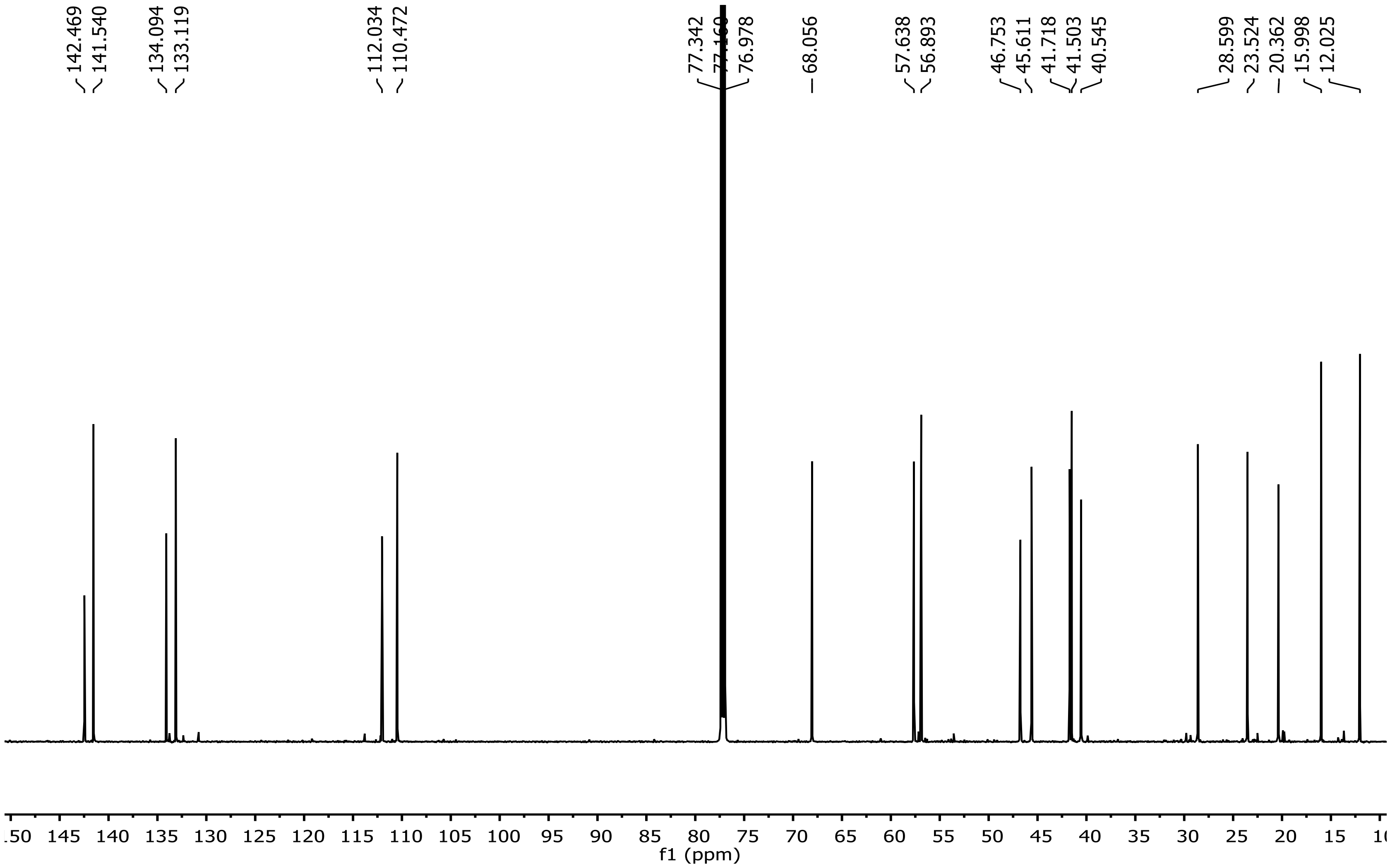

Figure S9. ^{13}C NMR ($CDCl_3$, 175 MHz) spectrum of **2**

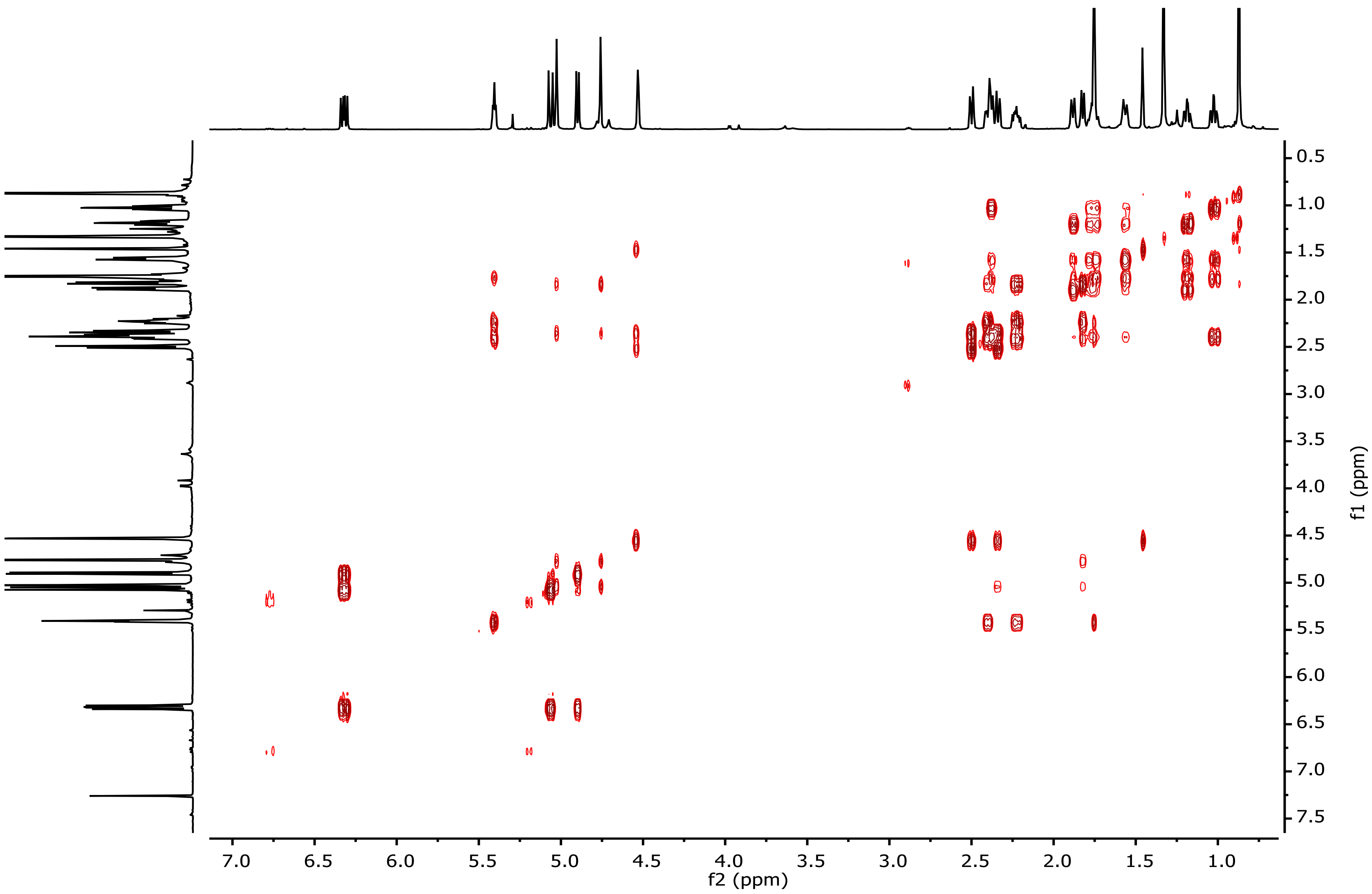

Figure S10. COSY NMR ($CDCl_3$, 700 MHz) spectrum of **2**

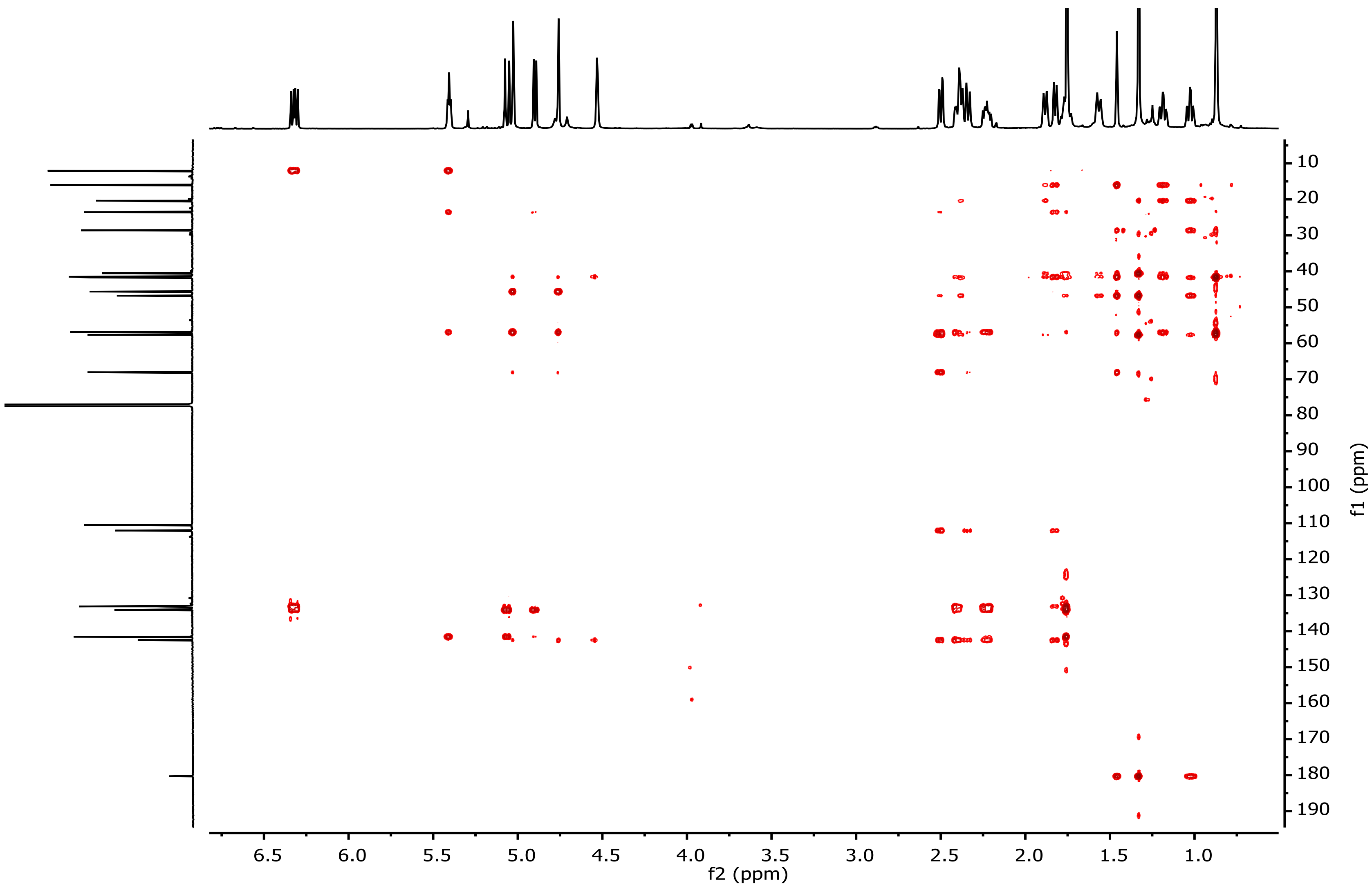

Figure S11. HMBC NMR ($CDCl_3$, 700 MHz) spectrum of **2**

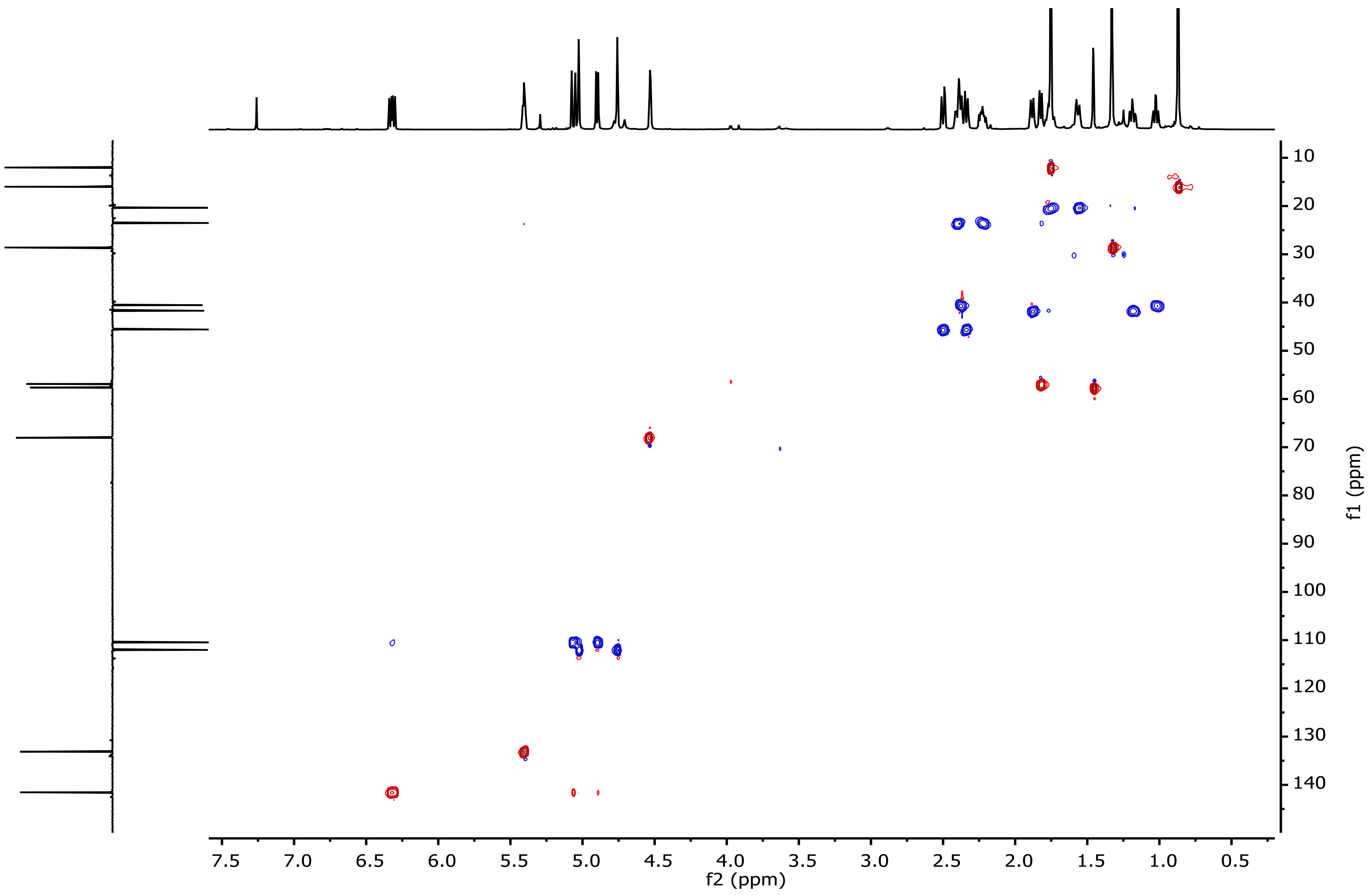

Figure S12. HSQC NMR ($CDCl_3$, 700 MHz) spectrum of **2**

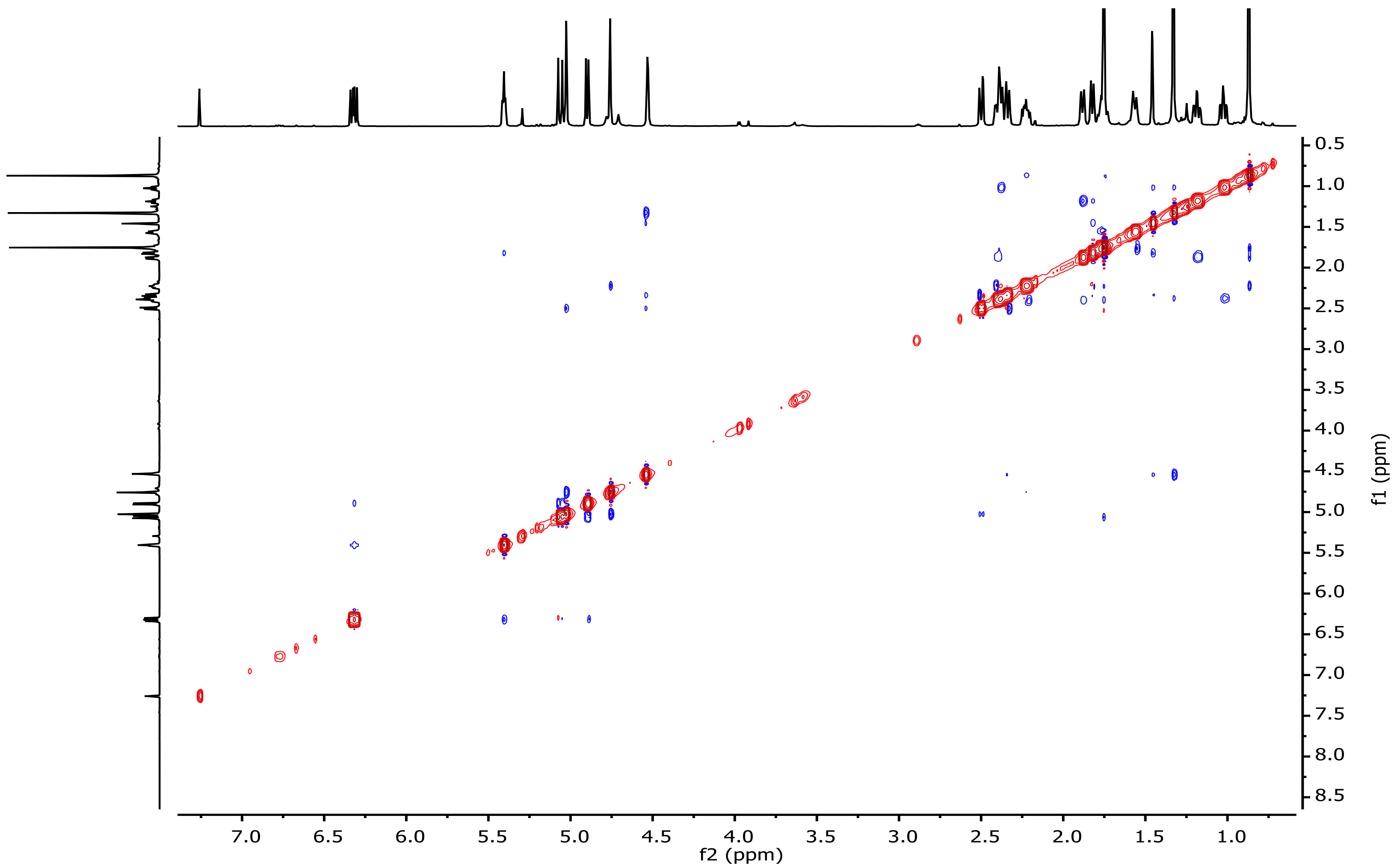

Figure S13. NOESY NMR ($CDCl_3$, 700 MHz) spectrum of **2**

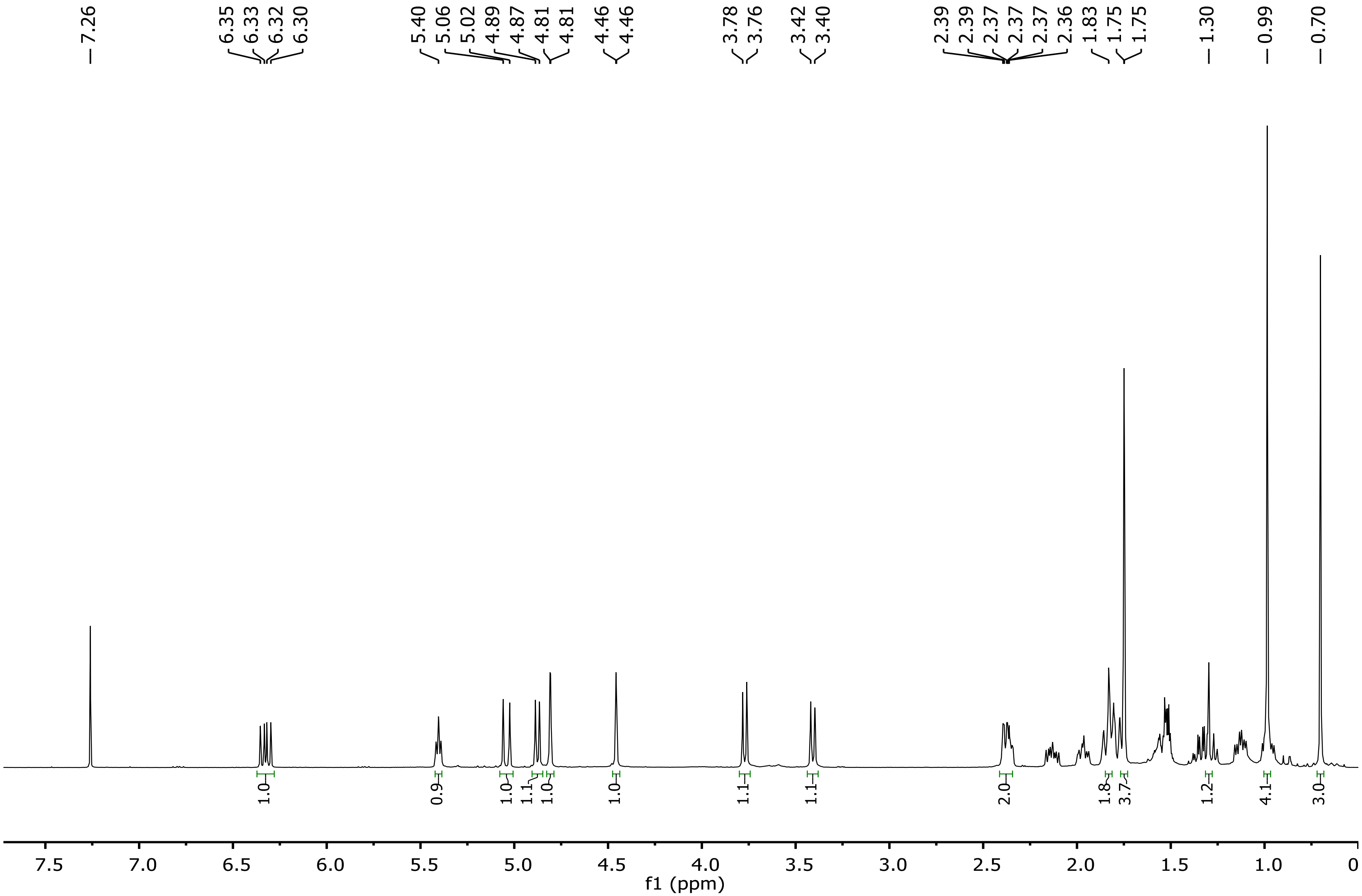

Figure S14. ^{1}H NMR ($CDCl_3$, 700 MHz) spectrum of **4**

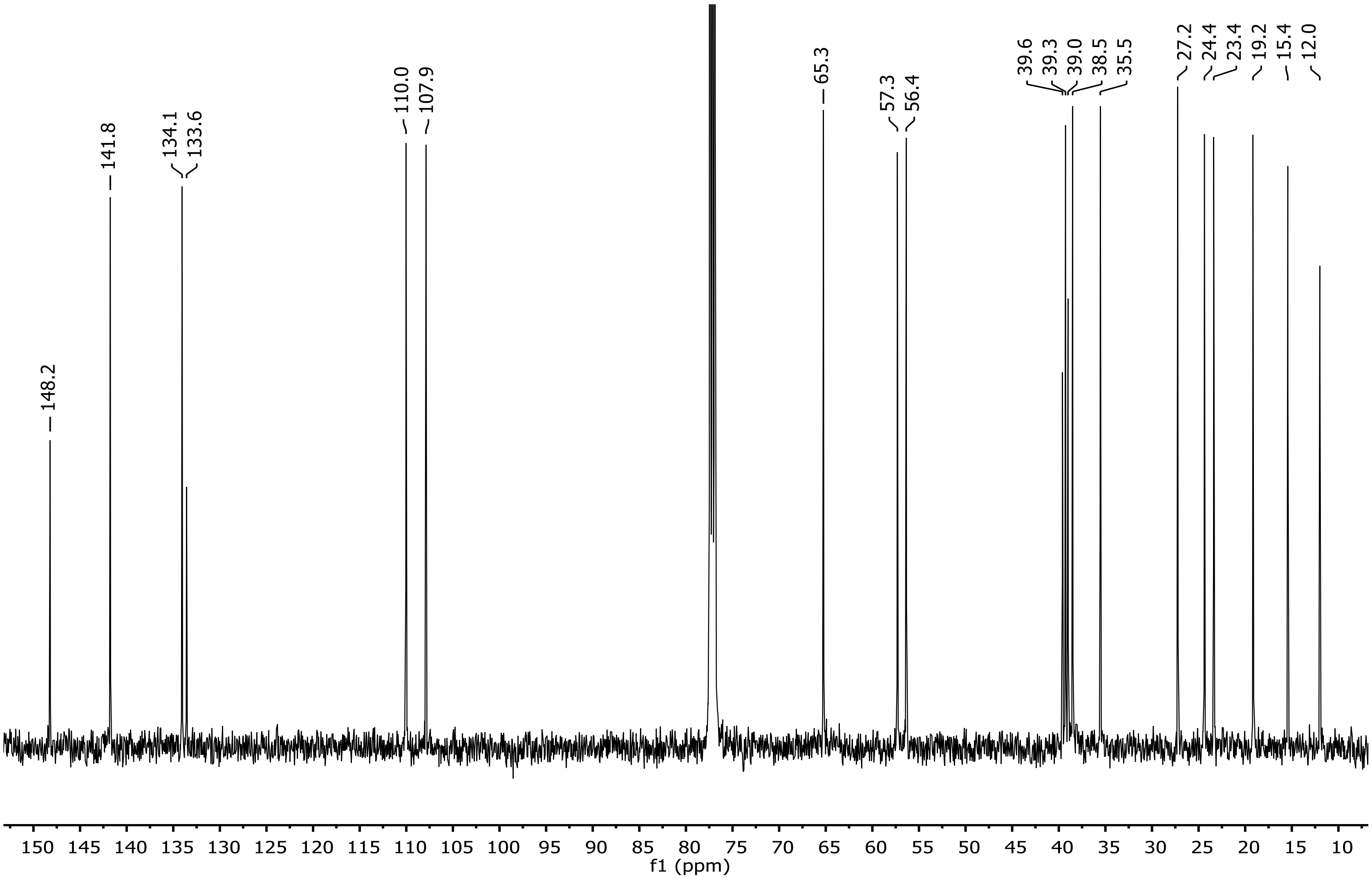

Figure S15. ^{13}C NMR ($CDCl_3$, 175 MHz) spectrum of **4**

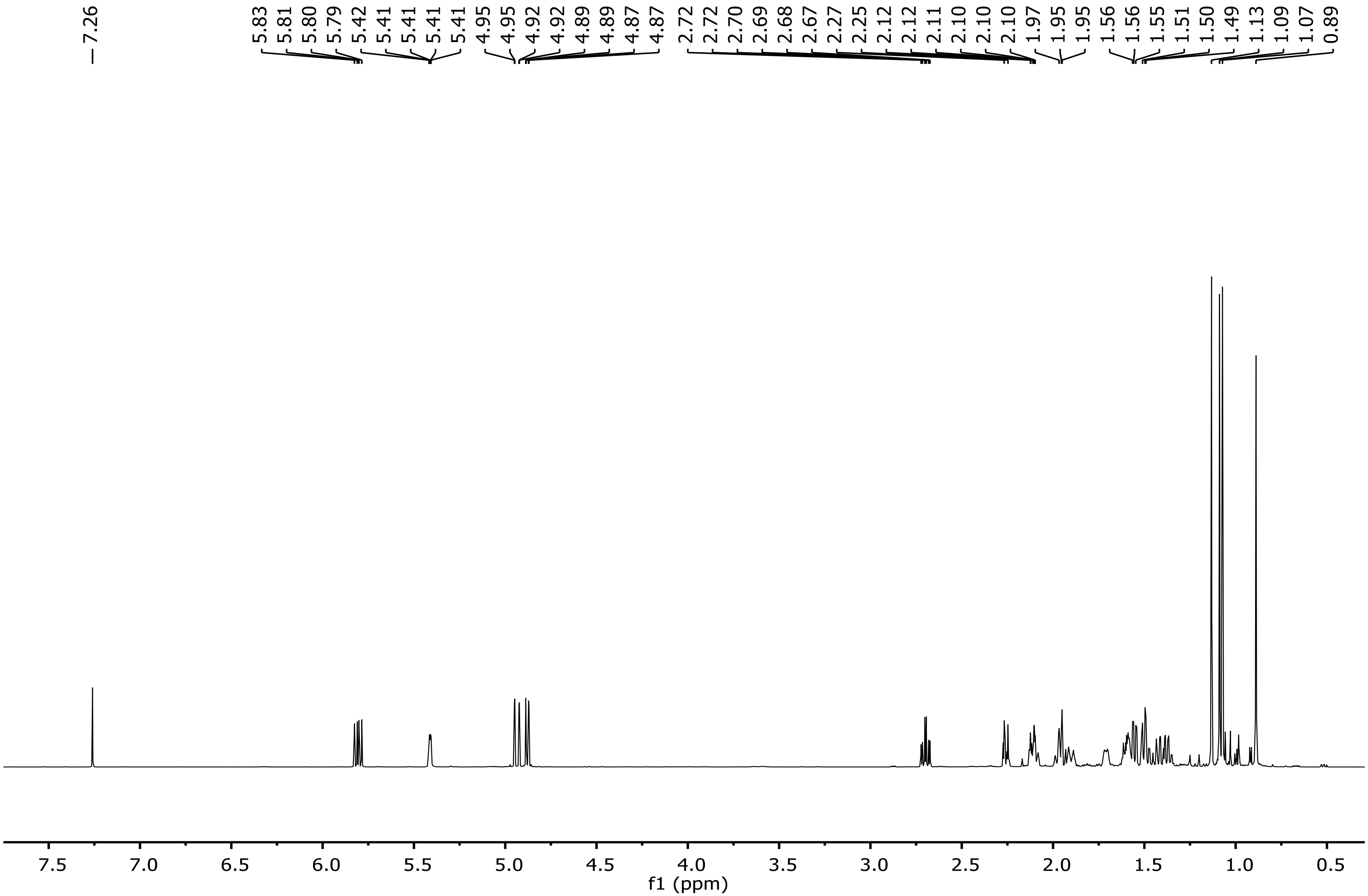

Figure S16. ¹H NMR ($CDCl_3$, 700 MHz) spectrum of **5**

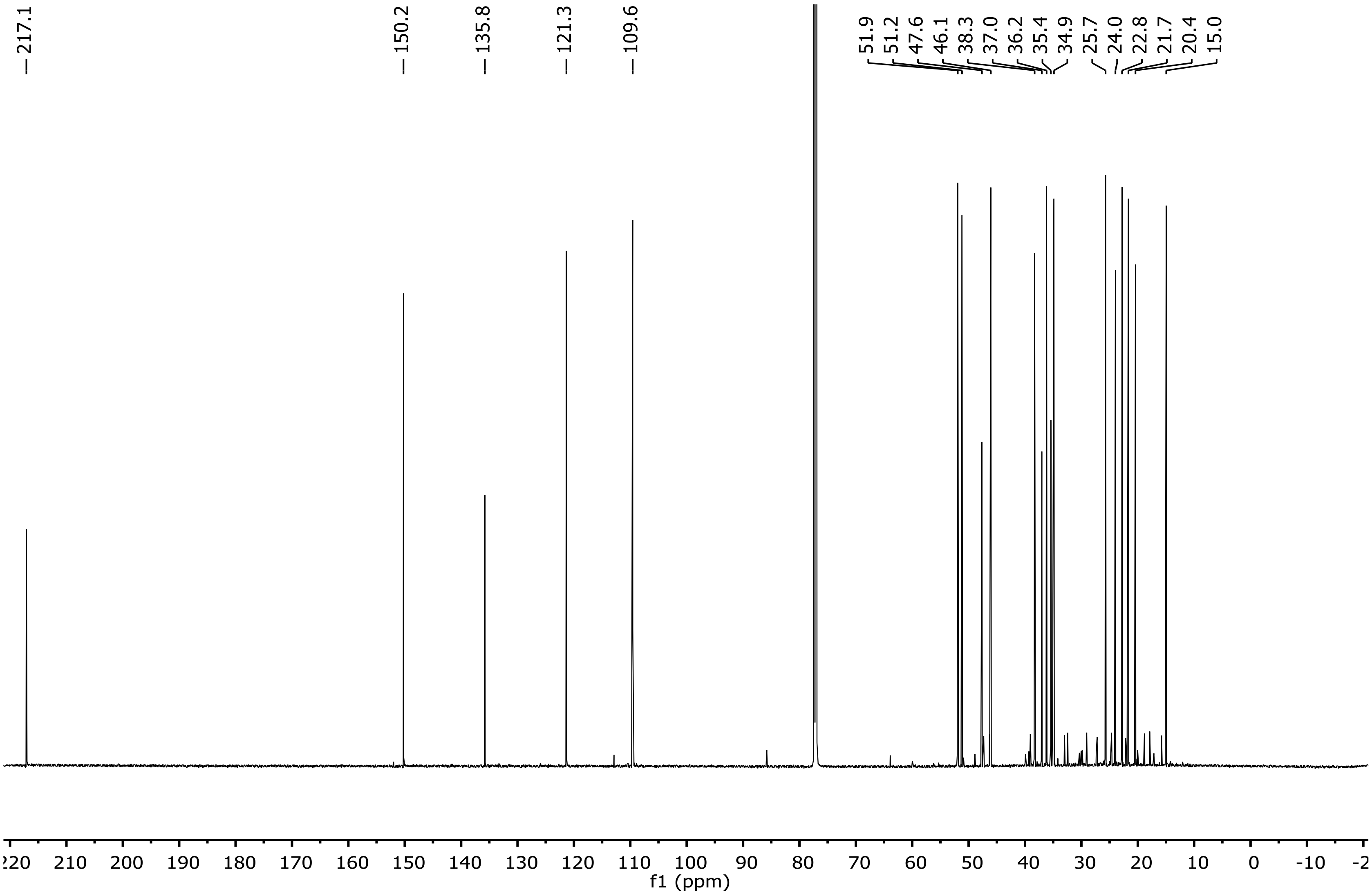

Figure S17. ^{13}C NMR ($CDCl_3$, 175 MHz) spectrum of **5**

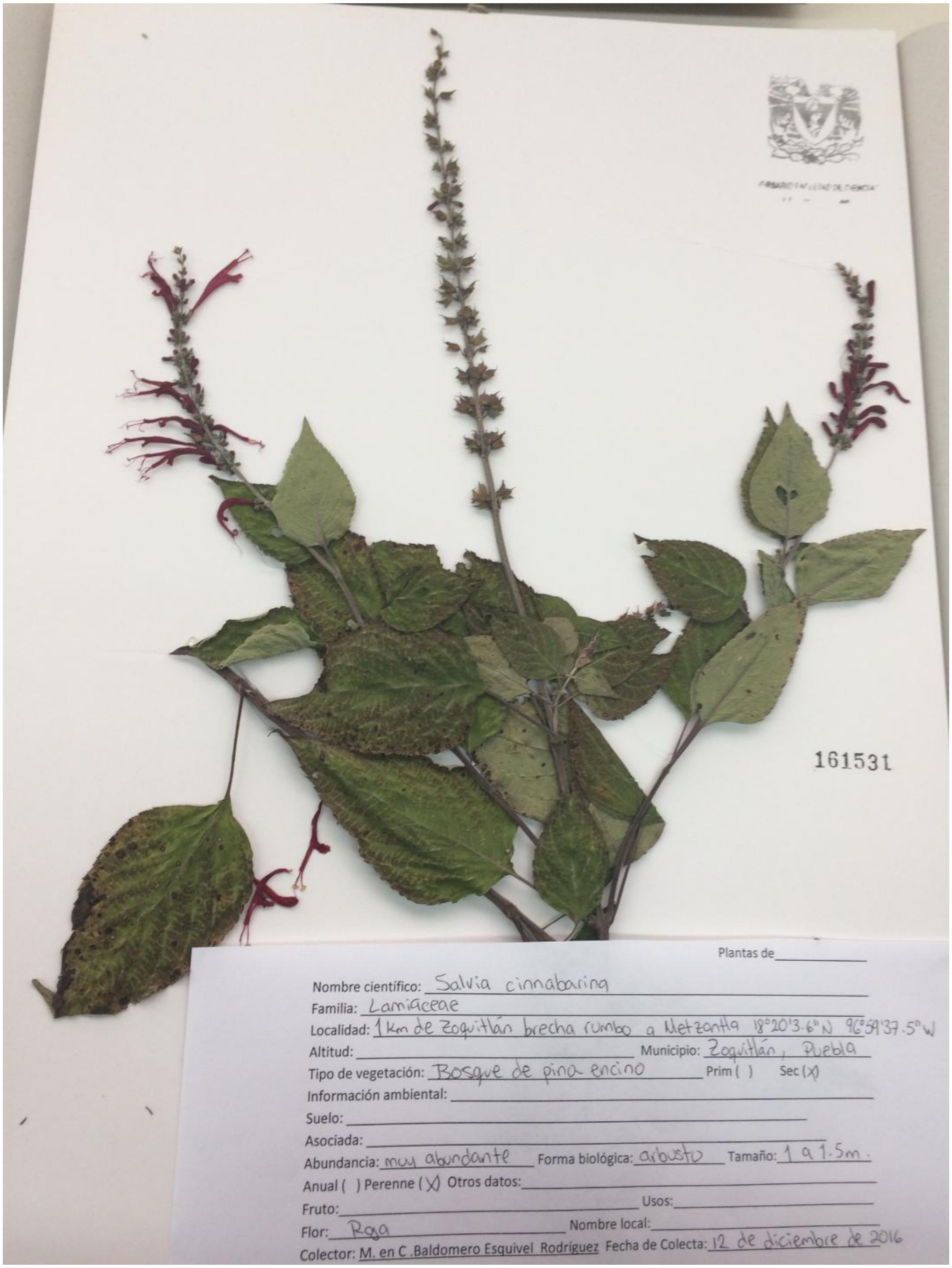

Figure S18. Herbarium specimen of *Salvia cinnabarina* collected by Dr. Baldomero Esquivel-Rodríguez collected in Zoquitlan, State of Puebla, Mexico, in December 2017.

Table S1. Primary screening of the Inhibitory effect of compounds **2**, **3** and **7** on TPA-induced inflammation in a mouse model.

**Muestra**	**Edema (mg)**	**Inhibición (%)**	**DI_{50} (μmol/oreja)**
Control (ethanol)	13.20±0.31	-	-
6-β-hidroxy-trans communico (**2**)	14.27±1.11	9.51 ± 7.08	ND
Trans communic acid (**3**)	10.33±1.03	21.72 ± 7.84	ND
β-eudesmol (**7**)	12.00±2.01	9.09 ± 15.25	ND

Effects on ear edema of female mice CD-1; Doses (1.0 μmol ear^{-1}); each value represents the mean of three animals ± SEM; The results were analyzed with the Student´s t test; The values at $p \leq 0.05$ (*) and $p \leq 0.01$ (**) were considered as significant differences with respect to the control group. ND = the compound was considered not active and the DI_{50} was no determined.

Table **S2**. Primary screening of inhibition of mammalian α-glucosidase activity for compounds **1** and **2**.

Compound	Concentration (μM)	Inhibition (%)	IC_{50}
**1**	1	2.91	ND
	10	3.46	
	100	2.48	
**2**	1	0.92	ND
	10	2.57	
	100	2.73	
Acarbose	1	25.84**	
	10	44.12**	
	100	56.88**	

Each value represents the mean of three independent experiments ± SEM; The data were analyzed by ANOVA followed by Dunnet post hoc test for comparison with control group. The values at $p \leq 0.05$ (*) and $p \leq 0.01$ (**) were considered as significant differences with respect to the control group. ND = the compound was considered not active and the IC_{50} was no determined.
